# CDC42‐Effector Proteins Regulate Higher Order Structure of Septins Required for CNS Myelin Integrity

**DOI:** 10.1002/glia.70134

**Published:** 2026-01-06

**Authors:** Sophie Hümmert, Joana Paes de Faria, Olaf Jahn, Ege Bilgin, Nikola Łukasik, Chethana Rao, Mišo Mitkovski, Fritz Benseler, Nils Brose, Sophie B. Siems, Sandra Goebbels, Wiebke Möbius, Helge Ewers, João B. Relvas, Hauke B. Werner

**Affiliations:** ^1^ Department of Neurogenetics Max Planck Institute for Multidisciplinary Sciences Göttingen Germany; ^2^ Instituto de Investigação e Inovação em Saúde (i3S), Universidade do Porto Porto Portugal; ^3^ Neuroproteomics Group, Department of Molecular Neurobiology Max Planck Institute for Multidisciplinary Sciences Göttingen Germany; ^4^ Translational Neuroproteomics Group, Department of Psychiatry and Psychotherapy University Medical Center Göttingen Göttingen Germany; ^5^ Institut für Chemie und Biochemie, Freie Universität Berlin Berlin Germany; ^6^ City Campus Light Microscopy Facility, Max Planck Institute for Multidisciplinary Sciences Göttingen Germany; ^7^ Department of Molecular Neurobiology Max Planck Institute for Multidisciplinary Sciences Göttingen Germany; ^8^ Cluster of Excellence ‘Multiscale Bioimaging: From Molecular Machines to Networks of Excitable Cells’ (MBExC), University of Göttingen Göttingen Germany; ^9^ Department of Biomedicine Faculdade de Medicina, Universidade do Porto Porto Portugal; ^10^ Faculty for Biology and Psychology, University of Göttingen Göttingen Germany

**Keywords:** CDC42, CDC42EP1, CDC42EP2, myelin outfoldings, myelin pathology, myelination, oligodendrocyte, septins, white matter

## Abstract

The regular structure of CNS myelin requires specialized structural proteins, including septin filaments composed of subunits SEPTIN2, SEPTIN4, SEPTIN7, and SEPTIN8. These filaments scaffold the innermost non‐compacted myelin layer; their disruption causes pathological myelin outfoldings. However, the mechanisms that control myelin septin assembly are incompletely understood. We found that loss of CDC42 from oligodendrocytes of adult mice causes myelin pathology including outfoldings, coinciding with depletion of myelin septins and CDC42‐effector proteins (CDC42EP1 and CDC42EP2). We thus tested the functional relevance of the latter by deleting both the *Cdc42ep1* and *Cdc42ep2*‐genes in oligodendrocytes. We observed myelin outfoldings as a very specific pathology, markedly reduced abundance of myelin septins, and disorganized septin filaments in myelin. Immunohistochemical analysis did not uncover astrocyte or microglial activation, implying that myelin outfoldings per se do not induce secondary neuropathology. Together, our data reveal a critical function for CDC42 and CDC42EP1/CDC42EP2 in regulating myelin septin filaments, which facilitate structural integrity of myelin sheaths.

## Graphical abstract

- CDC42‐effector proteins 1/2 are present in CNS myelin.
- They facilitate the higher order structure of myelin septin filaments.
- Their loss impairs septin‐dependent scaffolding of myelin.
- Myelin outfoldings do not cause secondary neuropathology per se.

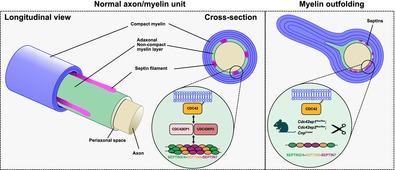

## 1 Introduction

In vertebrates, saltatory nerve conduction is achieved by restricting action potentials to short, specialized axonal segments, the nodes of Ranvier (Hartline and Colman 2007; Tasaki 1939). The long internodal segments of axons are electrically insulated by myelin sheaths, which in the central nervous system (CNS) are provided by oligodendrocytes (Bunge et al. 1962). Additionally, oligodendrocytes maintain axonal functions via extracellular vesicles, metabolic support, ion homeostasis, and antioxidant defense (Krämer‐Albers and Werner 2023). Mature myelin sheaths consist of multiple layers of specialized oligodendroglial plasma membrane with an unusually high proportion of lipids (70%–85%). Yet, healthy myelin sheaths require a particular composition of both myelin‐enriched lipids (Coetzee et al. 1996; Dimas et al. 2019; Saher et al. 2005; Wu et al. 2024) and specialized myelin proteins. This is exemplified by proteolipid protein (PLP), a cholesterol‐associated tetraspan transmembrane protein (Simons et al. 2000; Werner et al. 2013). PLP‐deficient mice, a genuine model of spastic paraplegia type‐2 (SPG2) (Garbern et al. 2002), display complex neuropathology that includes progressive axonopathy, neuroinflammation, hypomyelination, impaired myelin stability, and pathological outfoldings of myelin sheaths (Griffiths et al. 1998; Lüders et al. 2018; Patzig et al. 2016).

Myelin outfoldings, sometimes also referred to as focal hypermyelination, are a morphological abnormality of myelin sheaths presenting as compacted myelin layers excessively growing away from their adaxonal (innermost) non‐compacted myelin layer (Steyer et al. 2022). They are commonly observed in the context of complex neuropathology, as exemplified by mouse models of spastic paraplegia, for example, mice lacking PLP or myelin‐associated glycoprotein (MAG) (Biffiger et al. 2000; Li et al. 1994; Steyer et al. 2022). Myelin outfoldings are also a prominent feature of normal brain aging (Hill et al. 2018; Peters 2002; Safaiyan et al. 2016; Yassa 2014). In mice lacking PLP or MAG, as well as in aging mice, myelin outfoldings correlate with a reduced abundance of SEPTIN2, SEPTIN4, SEPTIN7, and SEPTIN8, and thus of all subunits of the septin filaments that scaffold the adaxonal myelin layer (Patzig et al. 2016). Indeed, disruption of myelin septins in mice causes myelin outfoldings as a highly specific neuropathology (Erwig et al. 2019b; Patzig et al. 2016). However, the factors that regulate myelin septin filaments are incompletely understood.

Septins are a family of small, filament‐forming GTP‐binding proteins (Mostowy and Cossart 2012) originally described in the context of cell division of budding yeast (Hartwell et al. 1973). Since then, septins have been identified in almost all eukaryotic clades, including humans (Shuman and Momany 2022). The 13 mammalian septin genes are categorized into four homology groups (Shuman and Momany 2022). Monomeric subunits polymerize into heterooligomers, usually hexamers or octamers, which may assemble into higher‐order structures, such as septin rings or longer filaments (Cavini et al. 2021), with functions ranging from interactions with other cytoskeletal elements (Kinoshita et al. 2002; Spiliotis and Nakos 2021), via formation of diffusion barriers and the control of membrane curvature and rigidity (Tanaka‐Takiguchi et al. 2009), to scaffolding (Spiliotis et al. 2005).

Septin filaments that scaffold CNS myelin (Patzig et al. 2016) share septin‐associated proteins with septin filaments relevant for other cellular functions in other cell types. This is exemplified by the cytoskeletal adaptor protein anillin (ANLN), which regulates cytokinesis at the completion of cell division (Naydenov et al. 2021). In post‐mitotic oligodendrocytes, ANLN facilitates septin filament assembly in the adaxonal myelin layer, thereby preventing the formation of myelin outfoldings (Erwig et al. 2019b). Considering that septins and ANLN evolved before myelin, it is plausible that, during myelin evolution, ANLN‐dependent septin assembly was recruited from other cellular functions.

The present study emerged from the hypothesis that the small Rho‐GTPase CDC42 and CDC42‐effector proteins expressed in oligodendrocytes affect myelin septin filaments and thus regulate myelin structure. Interactions between CDC42 and CDC42‐effector proteins (CDC42EP; also termed Binder of Rho‐GTPases (BORG)) affecting the higher order structure of septins were previously reported in other cell types that constitute septin filaments with different subunit composition. This is exemplified by CDC42‐dependent functions of CDC42EP5 in organizing SEPTIN2/SEPTIN6/SEPTIN7 multimers in MDCK epithelial cells (Joberty et al. 2001), of CDC42EP4 for multimers containing SEPTIN2, SEPTIN4, and SEPTIN7 in Bergmann glial cells (Ageta‐Ishihara et al. 2015), and of CDC42EP4 in SEPTIN7‐containing filaments in hematopoietic stem cells (Kandi et al. 2021). However, it remained unknown whether CDC42EP family members are present in oligodendrocytes and myelin, and, if so, whether they affect myelin septins. Our analysis was additionally motivated by the previous finding that mice lacking CDC42‐expression in oligodendrocytes display myelin outfoldings, including during development (Thurnherr et al. 2006). This effect of CDC42‐loss on myelin structure is similar to the myelin pathology seen upon deficiency of myelin septin filaments (Patzig et al. 2016), but the functional link between CDC42 and myelin septins was not tested at that time (Thurnherr et al. 2006) because the latter had not yet been discovered. Our present data show that CNS myelin contains a pool of CDC42EP1 (also termed BORG5) and CDC42EP2 (BORG1), whose presence is dependent on CDC42 and which regulate the higher order structure of myelin septins required for healthy myelin sheaths.

## 2 Results

### 2.1 CDC42 and Its Effector Proteins CDC42EP1 and CDC42EP2 Are Present in Oligodendrocytes and CNS Myelin

**FIGURE 1:**
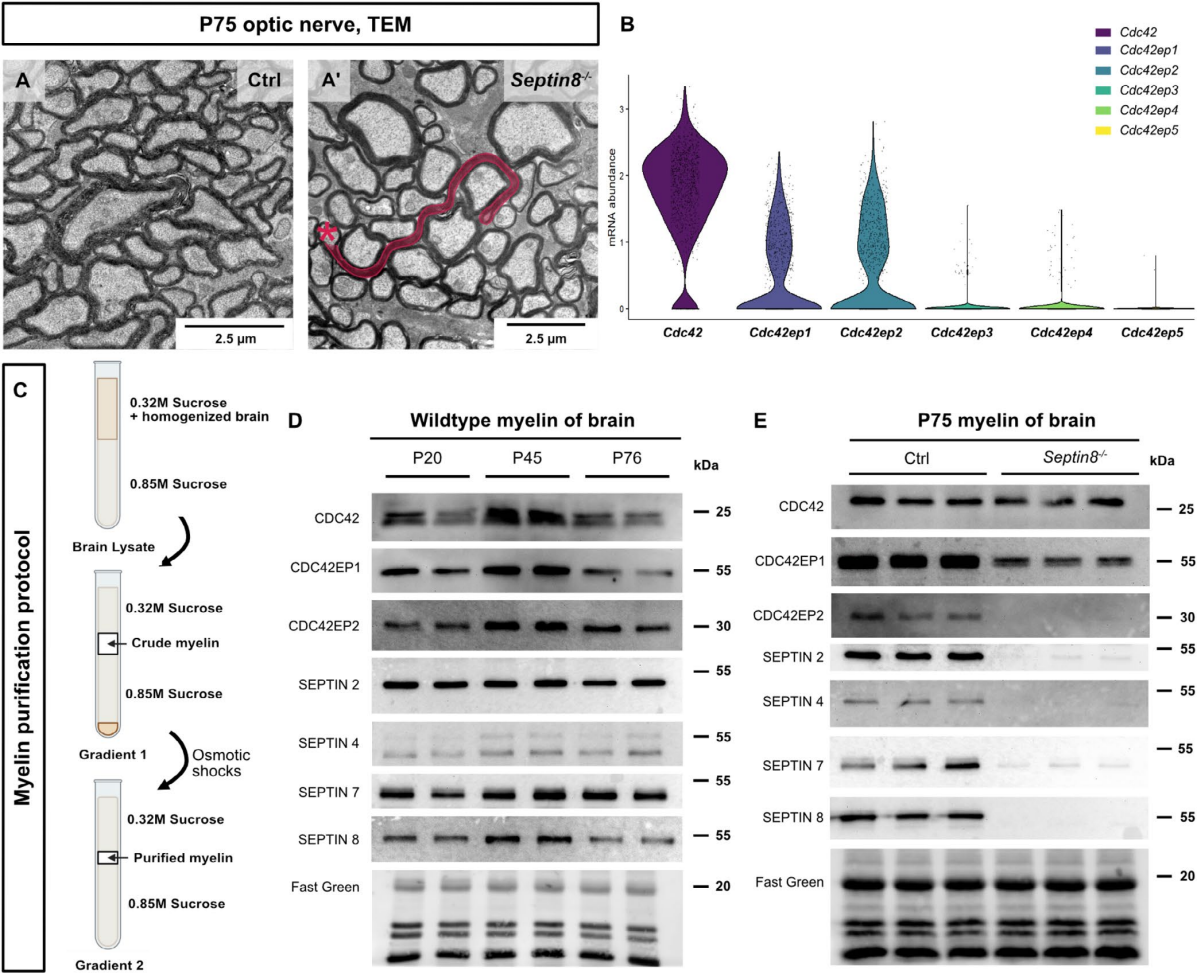
Septins, CDC42, and CDC42‐effector proteins CDC42EP1/CDC42EP2 in oligodendrocytes and myelin. (A, A′) Representative electron micrographs of optic nerves cross‐sectioned at P75 show that mice lacking *Septin8* (A′) but not control (A) mice develop pathological myelin outfoldings in the CNS, in agreement with prior quantitative assessment (Patzig et al. 2016). Myelin outfolding highlighted in red in (A′); asterisk indicates an axon associated with a myelin outfolding. TEM, transmission electron microscopy. (B) Violin plot of the abundance of the indicated transcripts in mature oligodendrocytes (MOL). Re‐analysis of previously published scRNA‐seq data (GSE75330; Marques et al. 2016). Each datapoint represents one out of 998 cells designated as mature oligodendrocytes (MOL; clusters MOL1‐MOL6 in Marques et al. 2016) in C57Bl/6 mice. Note that *Cdc42*, *Cdc42ep1*, and *Cdc42ep2* mRNAs are abundant in MOL when compared to *Cdc42ep3*, *Cdc42ep4*, and *Cdc42ep5* mRNAs. For bulk RNA‐seq data of oligodendrocytes immunopanned from mouse cortices (Zhang et al. 2014), see Figure S1. (C) Protocol of myelin purification via sucrose density gradient centrifugation and osmotic shocks. For details see (Erwig et al. 2019a). (D) Immunoblots detecting CDC42, CDC42EP1, CDC42EP2, and all myelin septin filament subunits (SEPTIN2, SEPTIN4, SEPTIN7, SEPTIN8) in myelin purified from brains of C57B/6 N‐mice at postnatal day 20 (P20), P45, and P76. Fast Green serves as loading control. *n* = 2 mice per age. (E) Immunoblotting reveals reduced abundance of CDC42EP1 and CDC42EP2 and validates diminishment of myelin septins in myelin purified from brains of *Septin8* ^*−/−*^ mice at P75 compared to controls (Ctrl). Fast Green as loading control. *n* = 3 mice per genotype.

Depleting septin filaments in CNS myelin by genetic disruption in *Septin8* ^−/−^ mice causes pathological myelin outfoldings as visualized by transmission electron microscopy (TEM) of cross‐sectioned optic nerves (Figure 1A,A′), in agreement with prior quantitative assessment (Patzig et al. 2016). Here we followed the hypothesis that myelin septin filaments are regulated by CDC42 and CDC42‐effector proteins. To test this hypothesis, we first assessed two previously published datasets to determine if oligodendrocytes express the corresponding mRNAs and, if so, which. *Cdc42*, *Cdc42ep1*, and *Cdc42ep2* mRNAs were readily detected in mature oligodendrocytes (MOL) both by scRNA‐seq (GEO dataset GSE75330; Marques et al. 2016; Figure 1B) and by bulk RNA‐Seq in cells designated as newly formed oligodendrocytes or myelinating oligodendrocytes sorted from the cortices of mice (GSE52564; Zhang et al. 2014; Figure 1). In contrast, *Cdc42ep3, Cdc42ep4*, and *Cdc42ep5* transcripts were detected at much lower levels.

We then biochemically purified myelin from mouse brains following an established protocol (Figure 1C; Erwig, et al. 2019a). CDC42, CDC42EP1, and CDC42EP2 were readily detected by immunoblotting (Figure 1D) at all assessed ages (postnatal day P20, P45, and P76) in myelin purified from brains of wildtype C57Bl/6N mice, as were the CNS myelin septin filament subunits (SEPTIN2, SEPTIN4, SEPTIN7, SEPTIN8) (Figure 1D). When assessing myelin purified from the brains of *Septin8* ^−/−^ and control mice by immunoblotting, SEPTIN2, SEPTIN4, SEPTIN7, and SEPTIN8 were virtually undetectable in *Septin8* ^−/−^ myelin (Figure 1E), in agreement with the previously reported (Patzig et al. 2016) disruption of myelin septin filaments. Interestingly, the abundance of CDC42EP1 was also markedly reduced and CDC42EP2 was virtually undetectable in *Septin8* ^−/−^ myelin (Figure 1E). Together these data indicate that CDC42 and its effector proteins CDC42EP1 and CDC42EP2 are expressed in oligodendrocytes, and that the presence of CDC42EP1 and CDC42EP2 in myelin depends on myelin septins.

### 2.2 Myelin Pathology Upon Deletion of *Cdc42* in Oligodendrocytes of Adult Mice

To test our hypothesis that CDC42 is involved in regulating myelin septin filaments, we used a previously generated mouse line with an allele of the *Cdc42* gene in which exon 2 is flanked by loxP sites (floxed) that is, *Cdc42* ^*flox*^ mice (Wu et al. 2006). *Cdc42* ^*flox*^ mice were previously used to delete *Cdc42* in myelinating oligodendrocytes, including during development (Thurnherr et al. 2006); *Cdc42* ^*flox/flox*^; *Cnp* ^*Cre*^ mice displayed myelin outfoldings (Thurnherr et al. 2006), and subsequent immunoblotting revealed reduced abundance of SEPTIN2 and SEPTIN8 in myelin purified from the brains of juvenile *Cdc42* ^*flox/flox*^; *Cnp* ^*Cre*^ mice (Paes De Faria et al. 2022).

**FIGURE 2:**
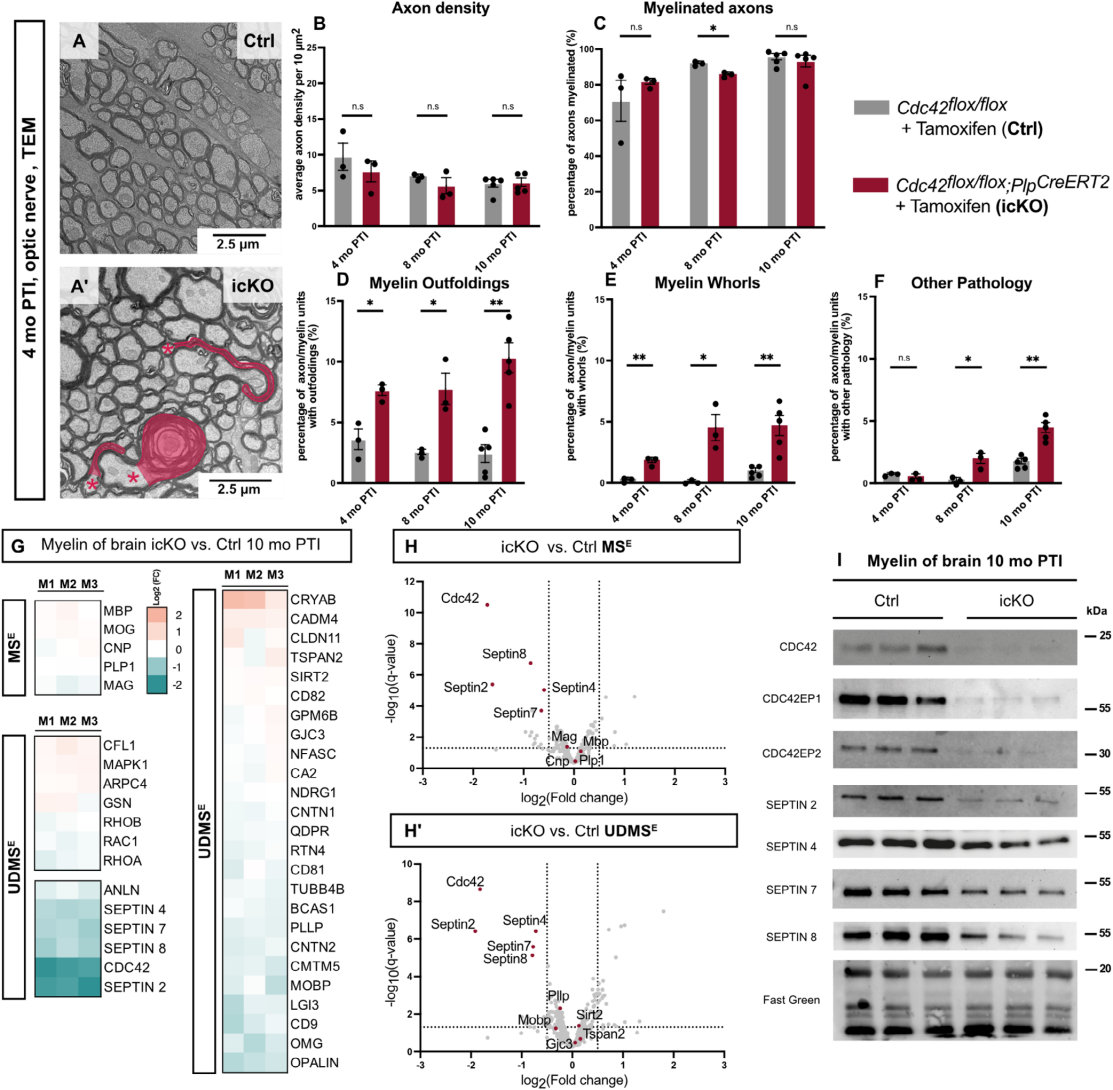
*Cdc42*‐deletion in oligodendrocytes of adult mice impairs myelin structure and alters protein composition. (A–F) Representative electron micrographs (A, A′) and genotype‐dependent quantification (B–F) of cross‐sectioned optic nerves showing myelin pathology in *Cdc42* ^*flox/flox*^;*Plp* ^*CreERT2*^ (icKO) compared to control (Ctrl) mice 4 months post tamoxifen injection (mo PTI). (A, A′) Myelin pathology highlighted in red; asterisks indicate associated axons. (B) Quantitative analysis of electron micrographs of optic nerves 4, 8, and 10 months PTI reveals normal axon density in *Cdc42*‐icKO mice. Mean ± SEM; datapoints represent individual mice; *n* = 3–5 mice; multiple unpaired t‐test with Holm‐Šídák correction (4 months PTI *p* = 0.441, 8 months PTI *p* = 0.282, 10 months PTI *p* = 0.875). C Quantitative analysis shows moderately but significantly reduced percentage of myelinated axons in *Cdc42*‐icKO mice 8 months PTI but not 4 or 10 months PTI. Mean ± SEM; datapoints represent individual mice; *n* = 3–5 mice; multiple unpaired t‐test with Holm‐Šídák correction (4 months PTI *p* = 0.400, 8 months PTI *p* = 0.005, 10 months PTI *p* = 0.537). D Quantitative analysis identifies increased percentage of axon/myelin‐units with myelin outfoldings in *Cdc42*‐icKO mice. Mean ± SEM; datapoints represent individual mice; *n* = 3–5 mice; multiple unpaired t‐test with Holm‐Šídák correction (4 months PTI *p* = 0.0135, 8 months PTI *p* = 0.0163, 10 months PTI *p* = 0.0005). (E) Quantitative analysis reveals increased percentage of axon/myelin‐units with myelin whorls in *Cdc42*‐icKO mice. Mean ± SEM; datapoints represent individual mice; *n* = 3–5 mice; multiple unpaired *t* test with Holm‐Šídák correction (4 months PTI *p* = 0.003, 8 months PTI *p* = 0.015, 10 months PTI *p* = 0.002). F Quantitative analysis shows increased percentage of axon/myelin‐units that display other pathology 8 and 10 months PTI. Mean ± SEM; datapoints represent individual mice; *n* = 3–5 mice; multiple unpaired *t* test with Holm‐Šídák correction (4 months PTI *p* = 0.263, 8 months PTI *p* = 0.017, 10 months PTI *p* = 0.0004). (G, H) Differential proteome analysis comparing the relative abundance of proteins in myelin purified from brains of *Cdc42*‐icKO and control (Crtrl) mice 10 months PTI. (G) Heatmap shows mass spectrometric quantification of known myelin constituents in three biological replicates (M1, M2, and M3) as the average of two technical replicates each, compared to the mean of Ctrl. Note that CDC42, myelin septins (SEPTIN2, SEPTIN4, SEPTIN7, SEPTIN8), and the septin‐associated adaptor protein anillin (ANLN) are diminished in myelin when oligodendrocytes lack *Cdc42*. (H, H′) Volcano plots summarizing genotype‐dependent quantitative myelin proteome analysis. Data points represent relative abundance of proteins quantified in myelin of *Cdc42*‐icKO compared to Ctrl mice 10 months PTI. Data points are plotted as log2‐transformed fold‐change on the x‐axis against the −log10‐transformed *q* value on the y‐axis according to two different data acquisition modes (see Methods section 4.3 for details) that is, MS^E^ (H; 391 proteins) and UDMS^E^ (B′; 535 proteins). The vertical stippled lines mark a log_2_‐fold change of 0.5 or −0.5 threshold of changed protein abundance in myelin, and the horizontal stippled line indicates a −log_10_‐transformed q‐value of 1.3 as significance threshold. Data points representing myelin septin subunits (SEPT2, SEPT4, SEPT7, and SEPT8) and CDC42 are highlighted in red with protein names given; their abundance is strongly reduced in *Cdc42*‐icKO compared to Ctrl myelin. Note that CDC42EP1 and CDC42EP2 are not identified by mass spectrometry in the present dataset. For dataset and exact *q* values see data Table S1. (I) Immunoblotting validates reduced abundance of CDC42 and myelin septins and reveals diminishment of CDC42EP1 and CDC42EP2 in myelin purified from *Cdc42*‐icKO mice 10months PTI. Fast Green as loading control. Blots show *n* = 3 mice per genotype.

To circumvent any potential confounding factors arising from the deletion of *Cdc42* during development, we here assessed the consequences of deleting the *Cdc42* gene in oligodendrocytes of juvenile mice by interbreeding *Cdc42* ^*flox*^ mice with *Plp* ^*CreERT2*^ mice (Leone et al. 2003). *Plp* ^*CreERT2*^ mice express tamoxifen‐inducible Cre‐recombinase (Feil et al. 1997) in myelinating oligodendrocytes, which allows temporal control of gene recombination. We obtained *Cdc42* ^*flox/flox*^; *Plp* ^*CreERT2*^ mice (also termed *Cdc42* icKO) and *Cdc42* ^*flox/flox*^ littermate controls (Ctrl). Both *Cdc42* icKO and Ctrl mice were injected with tamoxifen for five consecutive days starting at the juvenile age of 3 weeks. When using TEM to assess axon/myelin‐units in optic nerves of *Cdc42* icKO and Ctrl mice (Figure 2A), we found no genotype‐dependent differences in axonal density 4 months past tamoxifen injection (4 months PTI), 8 months PTI, and 10 months PTI (Figure 2B). The fraction of axons that are myelinated was unchanged 4 and 10 months PTI, but moderately reduced in *Cdc42* icKO mice 8 months PTI (Figure 2C). *Cdc42*‐dependent functions thus have a moderate effect on myelin maintenance in adult mice, which is in accordance with the previously reported moderate effect on developmental myelination in *Cdc42* ^*flox/flox*^; *Cnp* ^*Cre*^ mice (Thurnherr et al. 2006). However, the electron micrographs further revealed a considerable number of pathological axon/myelin‐profiles in *Cdc42* icKO mice. Quantitative evaluation showed a significantly increased frequency of pathological myelin outfoldings and myelin whorls in *Cdc42* icKO vs. Ctrl optic nerves at all tested timepoints PTI (Figure 2D,E). We also quantified further, infrequent pathological features (i.e., double myelination, lamella splittings, myelin inclusions, myelinoid bodies, signs of axonal pathology) jointly termed ‘other pathology'. The frequency of axon/myelin‐profiles with other pathology was increased 8 and 10 months PTI (Figure 2F). Myelin outfoldings in *Cdc42* icKO mice were not limited to optic nerves, as shown by TEM analysis of spinal cord (Figure S2A,B).

Next, we tested whether deletion of *Cdc42* in oligodendrocytes of juvenile mice affects the protein composition of myelin. By label‐free mass spectrometry, the relative abundance of most of the proteins quantified in myelin biochemically purified from the brains of *Cdc42* icKO mice 10 months PTI was unaltered compared to Ctrl mice. This includes myelin markers as PLP, MBP, MOG, CNP, and MAG, as well as the Rho‐GTPases Rac1, RhoA, and RhoB (Figure 2G,H; Table S1). As expected, the abundance of CDC42 was markedly reduced in *Cdc42* icKO myelin (Figure 2G,H,H′), in agreement with immunoblotting data (Figure 2I). Interestingly, the abundance of all myelin septin filament subunits (SEPTIN2, SEPTIN4, SEPTIN7, abd SEPTIN8) was also markedly reduced in *Cdc42* icKO compared to Ctrl myelin (Figure 2G,H), a finding that was validated by immunoblotting (Figure 2I). Although we have identified CDC42EP1 in our prior mouse CNS myelin proteome resource (Jahn et al. 2020), we consider CDC42EP1 and CDC42EP2 as myelin proteins of very low abundance prone to evade mass spectrometric identification, as seen here. We thus used immunoblotting to compare their abundance between *Cdc42* icKO and Ctrl myelin. Strikingly, both proteins were readily detected in Ctrl myelin but strongly reduced in *Cdc42* icKO myelin.

In summary, our analyses so far show that loss of CDC42 from oligodendrocytes of adult mice causes complex myelin pathology, including myelin outfoldings. This coincides with a striking reduction of CDC42EP1 and CDC42EP2 in CNS myelin of *Cdc42* icKO mice. It is plausible that myelin outfoldings in *Cdc42* icKO mice emerge owing to the diminishment of all subunits of the myelin septin filaments that scaffold CNS myelin (Patzig et al. 2016).

### 2.3 Myelin Pathology Upon Oligodendroglial Deletion of *Cdc42ep1* and *Cdc42ep2*

We next aimed to assess the functional relevance of CDC42EP1 and CDC42EP2 in myelin. Mice lacking CDC42EP1 (also termed BORG5) from all cells were generated previously (Liu et al. 2014), but analysis of *Cdc42ep1* ^*null/null*^ mice at that time was limited to microvasculature in embryonic heart development because of embryonic lethality, which also prevents analysis of the postnatal CNS. We thus generated mice harboring *Cdc42ep1* ^*flox*^ or *Cdc42ep2* ^*flox*^ alleles. By appropriate interbreedings with *Cnp* ^*Cre*^ mice expressing Cre recombinase in oligodendrocytes (Lappe‐Siefke et al. 2003), we achieved mice lacking CDC42EP1, or CDC42EP2, or both, specifically from oligodendrocytes that is, *Cdc42ep1* ^*flox/flox*^; *Cnp* ^*Cre*^ mice (also termed EP1 cKO), *Cdc42ep2* ^*flox/flox*^; *Cnp* ^*Cre*^ mice (also termed EP2 cKO), *Cdc42ep1* ^*flox/flox*^; *Cdc42ep2* ^*flox/flox*^; *Cnp* ^*Cre*^ mice (also termed dcKO), and respective control mice (also termed Ctrl).

**FIGURE 3:**
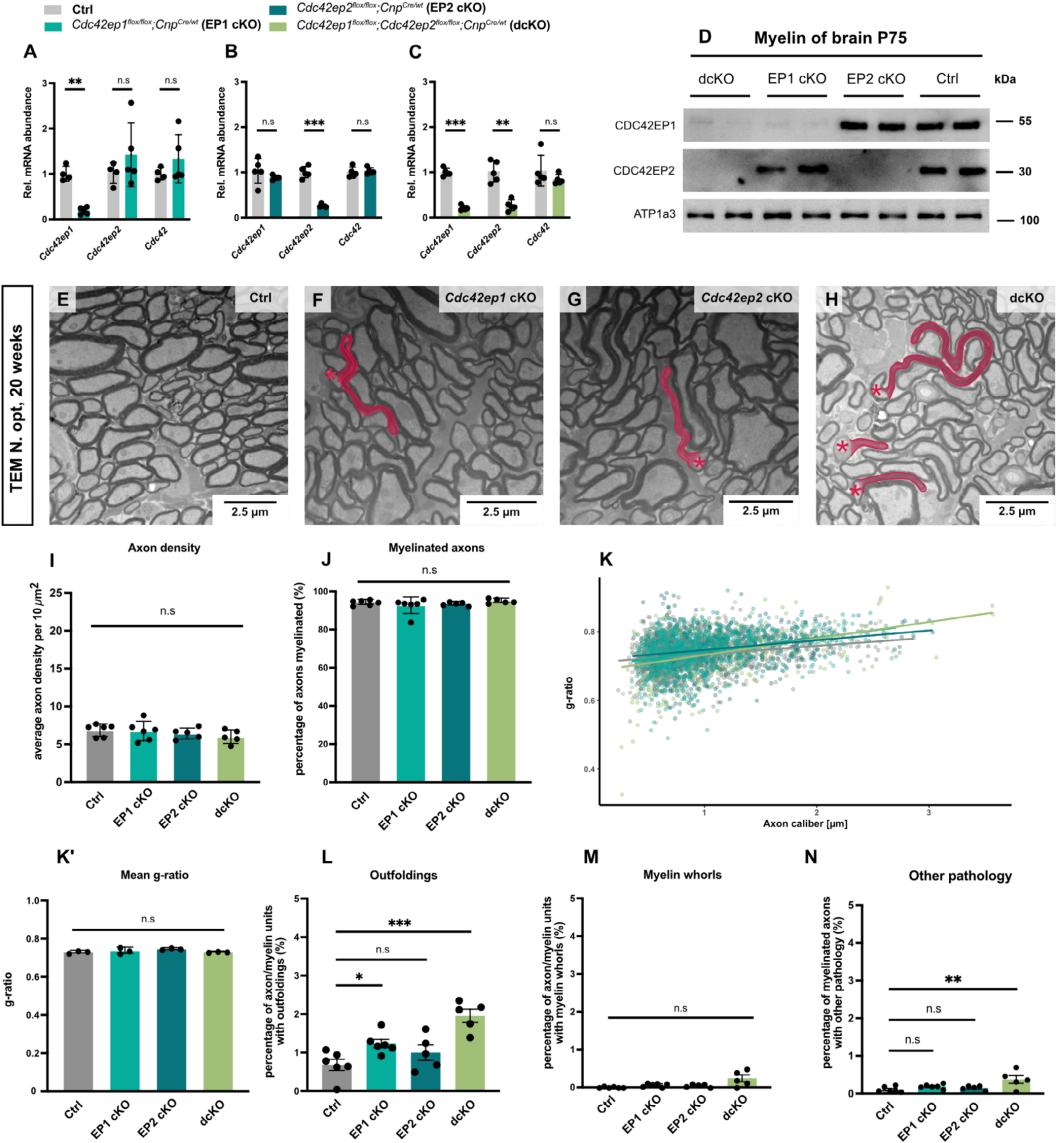
Myelination in mice upon deletion of *Cdc42ep1*, *Cdc42ep2*, or both genes in oligodendrocytes. (A–C) qRT‐PCR to determine the abundance of *Cdc42*, *Cdc42ep1*, and *Cdc42ep2* mRNAs in the white matter (corpora callosa) of *Cdc42ep1* ^*flox/flox*^; *Cnp* ^*Cre/wt*^ (EP1 cKO, A), *Cdc42ep2* ^*flox/flox*^; *Cnp* ^*Cre/wt*^ (EP2 cKO, B), *Cdc42ep1* ^*flox/flox*^; *Cdc42ep2* ^*flox/flox*^; *Cnp* ^*Cre/wt*^ (dcKO, C), and respective control (Ctrl) mice at age 20 weeks. *Cdc42ep1*‐mRNA was strongly reduced in EP1‐cKO and dcKO mice, *Cdc42ep2*‐mRNA was strongly reduced in EP2‐cKO and dcKO mice, while the abundance of *Cdc42*‐mRNA was unaltered. Mean ± SEM; data points represent individual mice; *n* = 4–5 mice. Data passed the Shapiro–Wilk test for normality and multiple unpaired t‐tests with parametric design; no assumption about consistent standard deviation. A *Cdc42ep1 p* = 0.0008, *Cdc42ep2 p* = 0.282, *Cdc42 p* = 0.246. (B) *Cdc42ep1 p* = 0.310, *Cdc42ep2 p* = 0.00009, *Cdc42 p* = 0.626. C *Cdc42ep1 p* = 0.000001, *Cdc42ep2 p* = 0.0008, *Cdc42 p* = 0.284. (D) Immunoblotting shows that CDC42EP1 is virtually undetectable in myelin purified from brains of EP1‐cKO or dcKO mice, and that CDC42EP2 is virtually undetectable in myelin purified from brains of EP2‐cKO or dcKO mice. Na^+^/K^+^‐transporting ATPase subunit‐α3 (ATP1a3) serves as loading control. Age P75; blots show *n* = 2 mice per genotype. (E–N) Representative electron micrographs (E–H) and genotype‐dependent quantification (I–N) of cross‐sectioned optic nerves reveal myelin outfoldings as the main pathology in *Cdc42ep1* ^*flox/flox*^; *Cdc42ep2* ^*flox/flox*^; *Cnp* ^*Cre/wt*^ (dcKO) mice at age 20 weeks. (E–H) Myelin outfoldings highlighted in red; asterisks indicate associated axons. I Quantification shows unchanged density of axons. Mean ± SEM; datapoints represent individual mice; *n* = 5–6 mice; one‐way ANOVA (Ctrl vs. EP1 cKO *p* = 0.994; Ctrl vs. EP2 cKO *p* = 0.805; Ctrl vs. dcKO *p* = 0.367). J Quantification reveals unchanged percentage of myelinated axons. Mean ± SEM; datapoints represent individual mice; *n* = 5–6 mice; one‐way ANOVA (Ctrl vs. EP1 cKO *p* = 0.486; Ctrl vs. EP2 cKO *p* = 0.945; Ctrl vs. dcKO *p* = 0.916). (K–K′) g‐ratio analysis indicates unchanged myelin sheath thickness. Datapoints represent axon/myelin‐units (K) or individual mice (K′); *n* = 3 mice; mean ± SEM (K′); one‐way ANOVA (Ctrl vs. EP1 cKO *p* = 0.726; Ctrl versus EP2 cKO *p* = 0.156; Ctrl vs. dcKO *p* > 0.999). (L) Quantification reveals increased percentage of axon/myelin‐units with myelin outfoldings in EP1 cKO and dcKO mice. *n* = 5–6 mice; mean ± SEM; one‐way ANOVA (Ctrl vs. EP1 cKO *p* = 0.044; Ctrl vs. EP2 cKO *p* = 0.372; Ctrl versus dcKO *p* < 0.0001). (M) Quantification shows unchanged percentage of axon/myelin‐units with myelin whorls. Mean +/− SEM; *n* = 5–6 mice; one‐way ANOVA (Ctrl vs. EP1 cKO *p* = 0.064; Ctrl vs. EP2 cKO *p* = 0.174; Ctrl versus dcKO *p* = 0.155). (N) Quantification reveals increased percentage of axon/myelin‐units displaying other pathology in dcKO mice. Mean ± SEM; datapoints represent individual mice; *n* = 5–6 mice; one‐way ANOVA (Ctrl vs. EP1 cKO *p* = 0.557; Ctrl vs. EP2 cKO *p* = 0.902; Ctrl vs. dcKO *p* = 0.006).

To validate the genetic disruption of the *Cdc42ep1* and *Cdc42ep2* genes in the newly generated mouse models, we amplified cDNA synthesized from a white matter tract (corpora callosa) dissected at 20 weeks of age (Figure 3A–C). We found a marked reduction of *Cdc42ep1* transcripts in EP1 cKO and dcKO corpora callosa (Figure 3A,C), and of *Cdc42ep2* transcripts in EP2 cKO and dcKO corpora callosa (Figure 3B,C). As a control, we amplified *Cdc42* mRNA, the relative abundance of which was similar in all genotypes. To examine if EP1 cKO, EP2 cKO, and dcKO mice display reduced abundance of the respective proteins in myelin, we performed immunoblot analyses of myelin biochemically purified from brains at P75. CDC42EP1 was virtually undetectable in EP1 cKO and dcKO myelin, and CDC42EP2 was virtually undetectable in EP2 cKO and dcKO myelin (Figure 3D). Taken together, these data show that oligodendroglial deletion of the *Cdc42ep1* and *Cdc42ep2* genes effectively eliminates expression of the respective protein products in myelin.

To assess consequences of the loss of CDC42EP1 and CDC42EP2 for the structure of the axon/myelin‐unit, we first analyzed optic nerves at 20 weeks of age by TEM (Figure 3E–H). Quantitative evaluation of electron micrographs revealed similar axonal density (Figure 3I), similar percentage of axons being myelinated (Figure 3J), and similar myelin sheath thickness (g‐ratio) (Figure 3K,K′) in EP1 cKO, EP2 cKO, and dcKO as compared to Ctrl mice. However, we noticed that the fraction of axon/myelin‐units displaying myelin outfoldings was considerably increased in EP1 cKO and dcKO mice (Figure 3L). EP2 cKO mice displayed a trend towards increased frequency of myelin outfoldings that did not reach significance. A trend towards increased frequency of myelin whorls in EP1 cKO and dcKO mice did not reach significance (Figure 3M). The frequency of other pathological features (double myelination, lamella splittings, myelin inclusions, myelinoid bodies, signs of axonal pathology) was moderately but significantly increased in dcKO but not in EP1 cKO or EP2 cKO mice (Figure 3N).

Together, these data show that mice lacking both CDC42EP1 and CDC42EP2 from oligodendrocytes display myelin outfoldings as a very specific pathology of the axon/myelin unit. In the following we largely focussed on analyzing dcKO and Ctrl mice, considering that our data imply that CDC42EP1 and CDCEP2 exert overlapping functions in oligodendrocytes.

**FIGURE 4:**
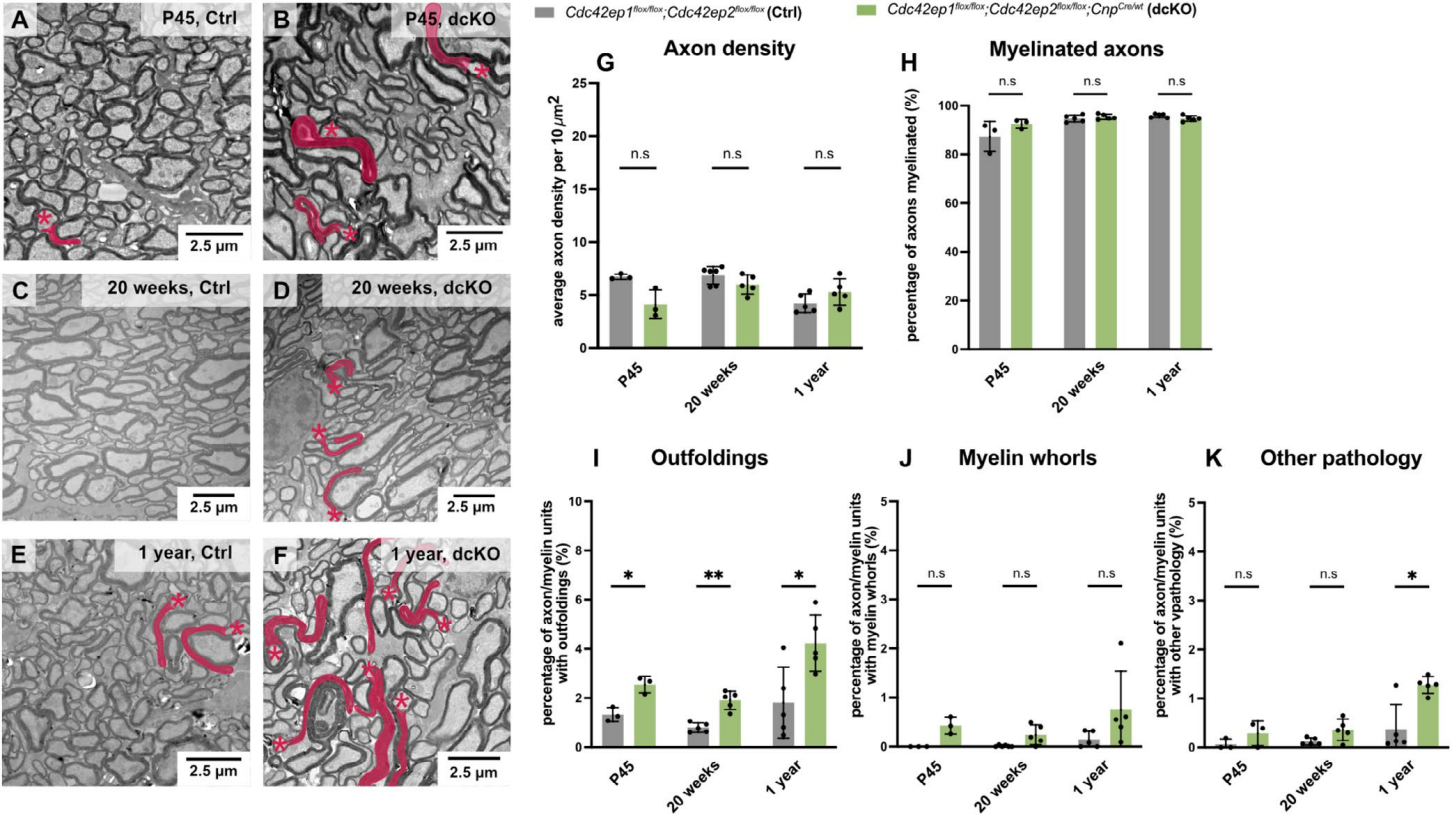
Progressive formation of myelin outfoldings when both *Cdc42ep1* and *Cdc42ep2* are lacking from oligodendrocytes. (A–K) Representative electron micrographs (A–F) and genotype‐dependent quantification (G–K) of cross‐sectioned optic nerves show that myelin outfoldings are the main pathology in *Cdc42ep1* ^*flox/flox*^; *Cdc42ep2* ^*flox/flox*^; *Cnp* ^*Cre/wt*^ (dcKO) mice. (A–F) Mice were analyzed at ages P45 (A, B), 20 weeks (C, D), and 1 year (E, F). Myelin outfoldings highlighted in red; asterisks indicate associated axons. (G) Quantification indicates unchanged density of axons. Mean ± SEM; datapoints represent individual mice; *n* = 3–5 mice; multiple unpaired *t* test with Holm‐Šídák correction (P45 *p* = 0.075, 20 weeks *p* = 0.142, 1 year *p* = 0.161). (H) Quantification shows unchanged percentage of myelinated axons. Mean ± SEM; datapoints represent individual mice; *n* = 3–5 mice; multiple unpaired *t* test with Holm‐Šídák correction (P45 *p* = 0.281, 20 weeks *p* = 0.393, 1 year *p* = 0.095). (I) Quantification reveals increased frequency of axon/myelin‐units with outfoldings in dcKO mice at ages P45, 20 weeks, and 1 year. Mean ± SEM; datapoints represent individual mice; *n* = 3–5 mice; multiple unpaired t‐test with Holm‐Šídák correction (P45 *p* = 0.001, 20 weeks *p* = 0.009, 1 year *p* = 0.019). (J) Quantification indicates unchanged percentage of axon/myelin‐units with myelin whorls. Mean ± SEM; datapoints represent individual mice; *n* = 3–5 mice; multiple unpaired *t* test with Holm‐Šídák correction (P45 *p* = 0.051, 20 weeks *p* = 0.066, 1 year *p* = 0.160). (K) Quantification reveals increased percentage of axon/myelin‐units with other pathology in dcKO mice at 1 year of age. Mean ± SEM; datapoints represent individual mice; *n* = 3–5 mice; multiple unpaired *t* test with Holm‐Šídák correction (P45 *p* = 0.261, 20 weeks *p* = 0.070, 1 year *p* = 0.013). (G‐K) Note that the data for Ctrl and dcKO at age 20 weeks are the same as in Figure 3I,J,L,M,N and are shown here for comparability.

To test how deletion of *Cdc42ep1* and *Cdc42ep2* in oligodendrocytes affects the structure of the axon/myelin‐unit over time, we assessed the optic nerves of dcKO and Ctrl mice by TEM at P45, 20 weeks, and 1 year of age (Figure 4A–F). Quantitative evaluation of electron micrographs revealed similar axonal density (Figure 4G) and similar percentage of myelinated axons in dcKO and Ctrl mice at all assessed ages (Figure 4H). However, the fraction of axon/myelin‐units with myelin outfoldings was considerably increased in dcKO mice (Figure 4I) at all assessed ages. A trend towards increased frequency of myelin whorls in dcKO mice did not reach significance (Figure 4J). The frequency of other pathological features of the axon/myelin‐unit was moderately but significantly increased in dcKO mice at 1 year of age (Figure 4K). We note that the data for Ctrl and dcKO mice at age 20 weeks in Figure 4G–K are the same as in Figure 3I,J,L,M,N and are plotted a second time to allow comparison with the other timepoints (P45, 1 year of age). At age 20 weeks, other pathological features displayed a trend towards increased frequency in dcKO compared to Ctrl mice that did not reach significance in the context of the comparison with other timepoints (in Figure 4K), while the same data showed a significant difference in the comparison with EP1 cKO or EP2 cKO mice (in Figure 3N). This discrepancy is explained by the use of different statistical tests with different correction methods, which lead to more conservative testing. Together, our data show that oligodendroglial deletion of *Cdc42ep1* and *Cdc42ep2* causes myelin outfoldings as a specific, moderately progressive pathology across multiple analyzed ages. The emergence of myelin outfoldings in dcKO mice was not limited to optic nerves, as shown by TEM‐analysis of spinal cords (Figure S2C,D).

**FIGURE 5:**
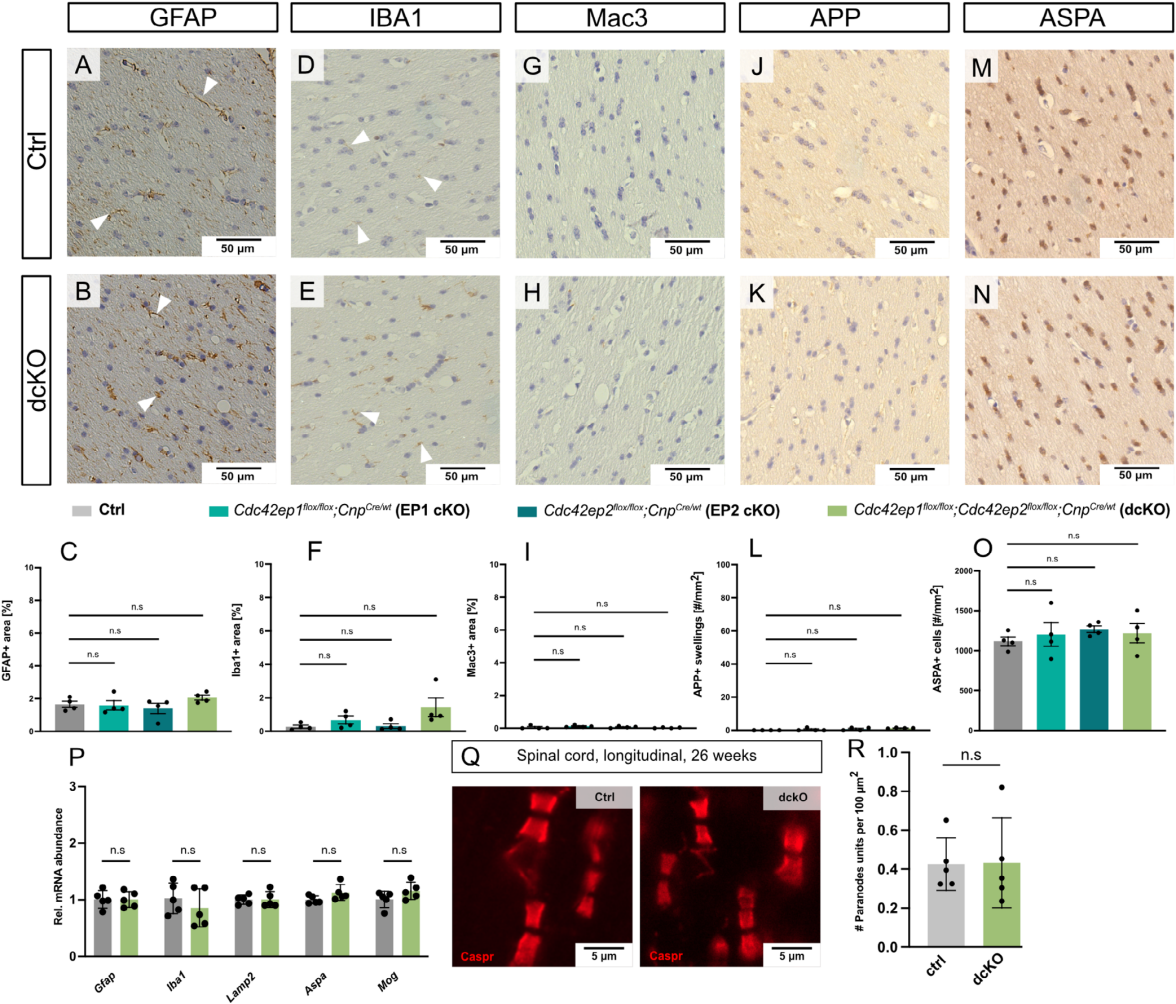
Neuropathological analysis of white matter upon oligodendroglial deletion of *Cdc42ep1*, *Cdc42ep2*, or both genes. (A–O) Representative light micrographs and genotype‐dependent quantification of secondary neuropathology. Shown are micrographs of a white matter tract (hippocampal fimbria) of *Cdc42ep1* ^*flox/flox*^; *Cdc42ep2* ^*flox/flox*^; *Cnp* ^*Cre/wt*^ (dcKO; B, E, H, K, and N) and control (Ctrl; A, D, G, J, and M) mice at age 1 year immunolabeled for the astrocyte marker GFAP (A, B), the microglia markers IBA1 (D, E) and MAC3/LAMP2 (G, H), the marker amyloid precursor protein (APP) to label axonal swellings (J,K), and the oligodendrocyte cell body marker ASPA (M,N). White arrowheads point at immunopositive cells. Scale bars 50 μm. (C) Quantification shows unchanged GFAP‐immunopositive area. Mean ± SEM; datapoints represent individual mice; *n* = 4 mice; one‐way ANOVA (Ctrl vs. EP1 cKO *p* = 0.996; Ctrl vs. EP2 cKO *p* = 0.820; Ctrl vs. dcKO *p* = 0.518). F Quantification shows unchanged IBA1‐immunopositive area. Mean +/‐SEM; datapoints represent individual mice; *n* = 4 mice; one‐way ANOVA (Ctrl vs. EP1 cKO *p* = 0.703; Ctrl vs. EP2 cKO *p* = 0.999; Ctrl vs. dcKO *p* = 0.057). I Quantification shows unchanged MAC3/LAMP2‐immunopositive area. Mean ± SEM; datapoints represent individual mice; *n* = 4 mice; Kruskal–Wallis test (Ctrl vs. EP1 cKO *p* = 247; Ctrl vs. EP2 cKO *p* > 0.999; Ctrl vs. dcKO *p* > 0.999). (L) Quantification shows unchanged number of APP‐immunopositive axonal swellings. Mean ± SEM; datapoints represent individual mice; *n* = 4 mice; Kruskal–Wallis test (Ctrl vs. EP1 cKO *p* > 0.999; Ctrl versus EP2 cKO *p* = 0.492; Ctrl vs. dcKO *p* = 0.065). (O) Quantification shows unchanged number of ASPA‐immunopositive oligodendrocytes. Mean ± SEM; datapoints represent individual mice; *n* = 4 mice; one‐way ANOVA (Ctrl vs. EP1 cKO *p* = 0.882; Ctrl vs. EP2 cKO *p* = 0.597; Ctrl vs. dcKO *p* = 0.823). (P) qRT‐PCR to determine the abundance of neuropathology associated (*Gfap*, *Iba1*, and *Lamp2*) and oligodendroglial (*Aspa* and *Mog*) transcripts in the white matter (corpora callosa) of dcKO and Ctrl mice at 20 weeks of age. Mean ± SEM; datapoints represent individual mice; *n* = 5 mice; data passed the Shapiro–Wilk test for normality; multiple unpaired t‐test with parametric design; no assumption about consistent SDs and *p* = 0.05 threshold for *p* value comparisons (*Gfap p* = 0.977, *Iba1 p* = 0.405, *Lamp2 p* = 0.949, *Aspa p* = 0.1149, *Mog p* = 0.145). (Q–R) Representative immunofluorescence labelling of the paranode marker CASPR on longitudinal spinal cord sections of *Cdc42ep1* ^*flox/flox*^; *Cdc42ep2* ^*flox/flox*^; *Cnp* ^*Cre/wt*^ (dcKO) and Ctrl mice (Q) to assess the density of paranodes, and genotype‐dependent quantification (R). R Quantification shows unchanged density of CASPR‐immunopositive paranodes. Mean ± SEM; datapoints represent individual mice; *n* = 5 mice; unpaired t‐test (Ctrl vs. dcKO *p* = 0.956).

Myelin pathology frequently coincides with secondary neuropathology (Lappe‐Siefke et al. 2003; Edgar et al. 2009; Lüders et al. 2017). To test if EP1 cKO, EP2 cKO, or dcKO mice develop astrogliosis, microgliosis, axonal pathology, or changes in oligodendrocyte numbers, we performed immunohistological analyses of the hippocampal fimbria as a representative white matter tract at 1 year of age (Figure 5A–O). Upon immunolabeling GFAP (Figure 5A–C) as a marker for astrocytes or IBA1 (Figure 5D–F) or MAC3 (also termed LAMP2; Figure 5G–I) as markers for microglia, the area of immunopositivity was similar in fimbriae of mice of all four genotypes. Similarly, when immunolabeling amyloid precursor protein (APP) as a marker for pathological axonal swellings (Figure 5J–L) or aspartoacylase (ASPA) as a marker for the cell bodies of myelinating oligodendrocytes (Figure 5M–O), we did not detect genotype‐dependent differences in their quantity.

Neuropathological assessment thus did not reveal signs of secondary neuropathology in EP1 cKO, EP2 cKO, or dcKO mice. This finding is in agreement with qRT‐PCR analyses, in which we amplified cDNA synthesized from a white matter tract (corpora callosa) dissected at 20 weeks of age and found that the relative abundance of *Gfap, Iba1, Lamp2/Mac3, Aspa*, and *Mog* transcripts was similar in dcKO and Ctrl mice (Figure 5P). Together, this shows that the presence of myelin outfoldings per se does not induce neuroinflammation.

To assess if the myelin outfoldings in dcKO mice coincide with an increased frequency of paranodes that could reflect reduced length of internodes, we immunolabeled longitudinally sectioned spinal cords dissected from dcKO and Ctrl mice at 26 weeks of age (Figure 5Q,R). Immunolabeling of contactin‐associated protein‐1 (CASPR) as a paranode marker (Figure 5Q) and quantification (Figure 5R) did not reveal genotype‐dependent differences in paranode density, and thus absence of evidence for altered internode length in dcKO mice.

### 2.4 Molecular Composition of Myelin When Oligodendrocytes Lack CDC42EP1 and CDC42EP2

**FIGURE 6:**
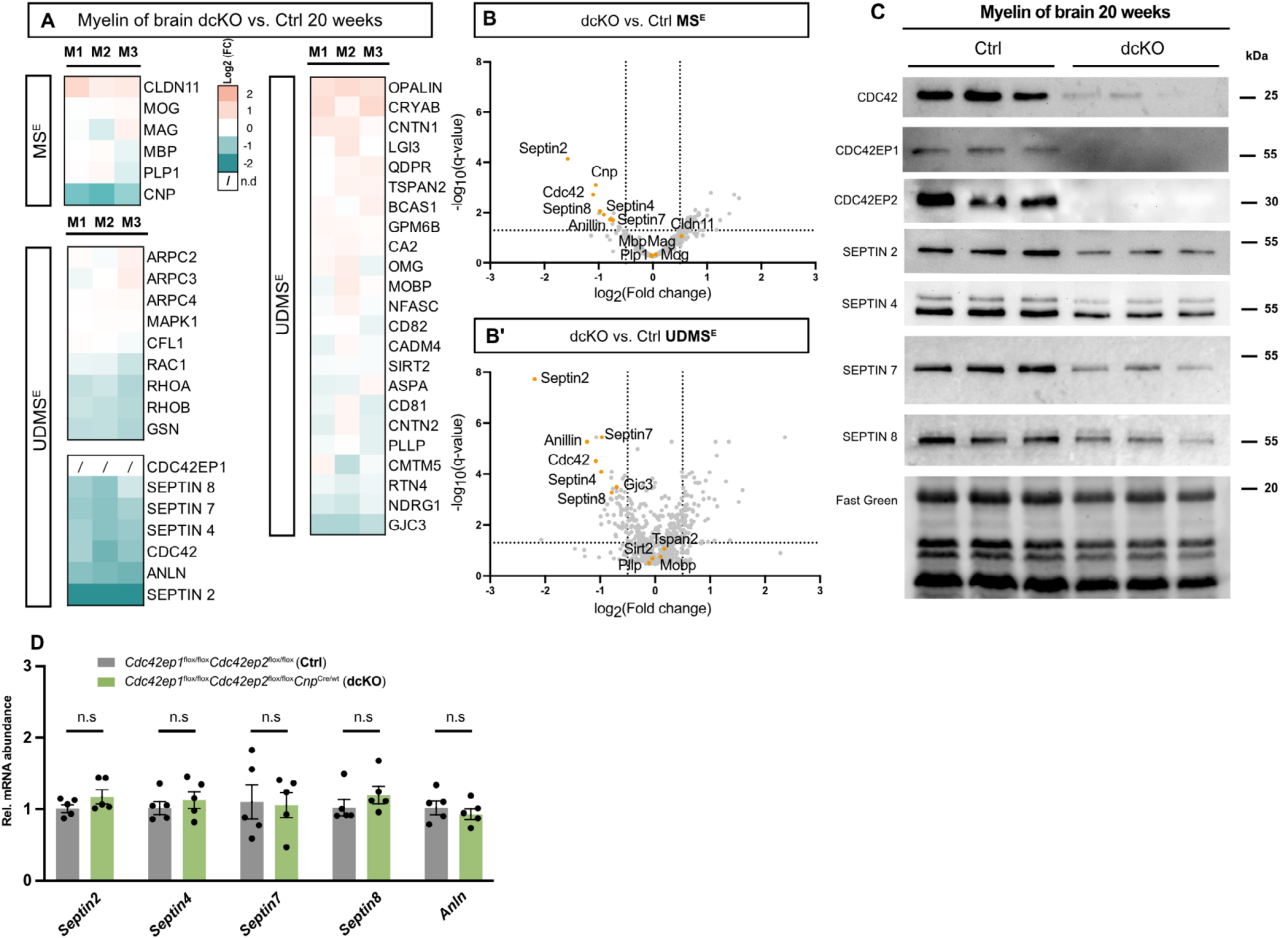
Myelin protein composition is altered when *Cdc42ep1* and *Cdc42ep2* are lacking from oligodendrocytes. (A, B) Differential proteome analysis comparing the relative abundance of proteins in myelin purified from brains of *Cdc42ep1* ^*flox/flox*^; *Cdc42ep2* ^*flox/flox*^ *Cnp* ^*Cre/wt*^ (dcKO) and control (Ctrl) mice at 20 weeks of age. A heatmap shows mass spectrometric quantification of known myelin constituents in three biological replicates (M1, M2, and M3) as the average of two technical replicates each, compared to the mean of Ctrl. Note that the abundance of myelin septins (SEPTIN2, SEPTIN4, SEPTIN7, and SEPTIN8), the septin‐associated adaptor protein anillin (ANLN), and CDC42 in myelin is reduced when oligodendrocytes lack *Cdc42ep1* and *Cdc42ep2*. (B) Volcano plots summarizing genotype‐dependent quantitative myelin proteome analysis. Data points represent relative abundance of proteins quantified in myelin of dcKO compared to Ctrl mice. Data points are plotted as log2‐transformed fold‐change on the x‐axis against the −log10‐transformed *q* value on the y‐axis according to two different data acquisition modes (see Section 4.3 for details) that is, MS^E^ (B; 459 proteins) and UDMS^E^ (B′; 728 proteins). The vertical stippled lines mark a log_2_‐fold change of 0.5 or −0.5 threshold of changed protein abundance in myelin, and the horizontal stippled line indicates a −log_10_‐transformed q‐value of 1.3 as significance threshold. Data points representing myelin septin subunits (SEPTIN2, SEPTIN4, SEPTIN7, SEPTIN8), ANLN, and CDC42 are highlighted in orange with protein names given; their abundance is strongly reduced in dcKO compared to Ctrl myelin. Note that CDC42EP1 is identified by mass spectrometry in EP2 cKO and Ctrl samples in the present UDMS^E^ data set; CDC42EP2 is not identified. For dataset and exact q‐values see data Table S2. (C) Immunoblot validates reduced abundance of myelin septins and CDC42 and virtual absence of CDC42EP1 and CDC42EP2 in myelin purified from *Cdc42ep1* ^*flox/flox*^; *Cdc42ep2* ^*flox/flox*^; *Cnp* ^*Cre/wt*^ (dcKO) mice compared to control (Ctrl) mice. Fast Green serves as loading control. (D) qRT‐PCR to determine the abundance of mRNAs encoding myelin septin subunits (*Septin2*, *Septin4*, *Septin7*, and *Septin8*) and the septin‐associated adaptor anillin (*Anln*) in the white matter (corpora callosa) of dcKO and Ctrl mice at 26 weeks of age. Mean ± SEM; datapoints represent individual mice; *n* = 5 mice per genotype. Data did not pass Shapiro–Wilk test for normality; unpaired Mann–Whitney test with nonparametric design and 0.05 threshold for *p* value comparisons (*Septin2 p* = 0.547, *Septin4 p* = 0.690, *Septin7 p* > 0.999, *Septin8 p* = 0.134, and *Anln p* = 0.547).

Next, we tested if oligodendroglial deletion of *Cdc42ep1* and *Cdc42ep2* affects the protein composition of myelin. By label‐free mass spectrometry, the relative abundance of most of the proteins quantified in myelin biochemically purified from the brains of dcKO mice at 20 weeks of age was unchanged compared to Ctrl, including that of the myelin markers PLP, MBP, MAG, MOG, and MOBP (Figure 6A,B,B′; Table S2). Notably, the most striking change was the reduced abundance of all myelin septin subunits (SEPTIN2, SEPTIN4, SEPTIN7, SEPTIN8) and of CDC42 in dcKO compared to Ctrl myelin (Figure 6A,B,B′), which was validated by immunoblotting (Figure 6C).

By qRT‐PCR, cDNA fragments for *Septin2*, *Septin4*, *Septin7*, *Septin8* (Figure 6D) and *Cdc42* (Figure 3C) were amplified with equal efficiency from dcKO and control corpora callosa, indicating that the reduced abundance of septins and CDC42 in dcKO myelin is a posttranscriptional event secondary to deficiency for CDC42EP1 and CDC42EP2. Together, these findings show that CDC42EP1 and CDC42EP2 are required for normal myelin protein composition, particularly for the presence of myelin septins and CDC42 in myelin.

### 2.5 Disorganized Myelin Septin Filaments When CDC42EP1 and CDC42EP2 Are Lacking

**FIGURE 7:**
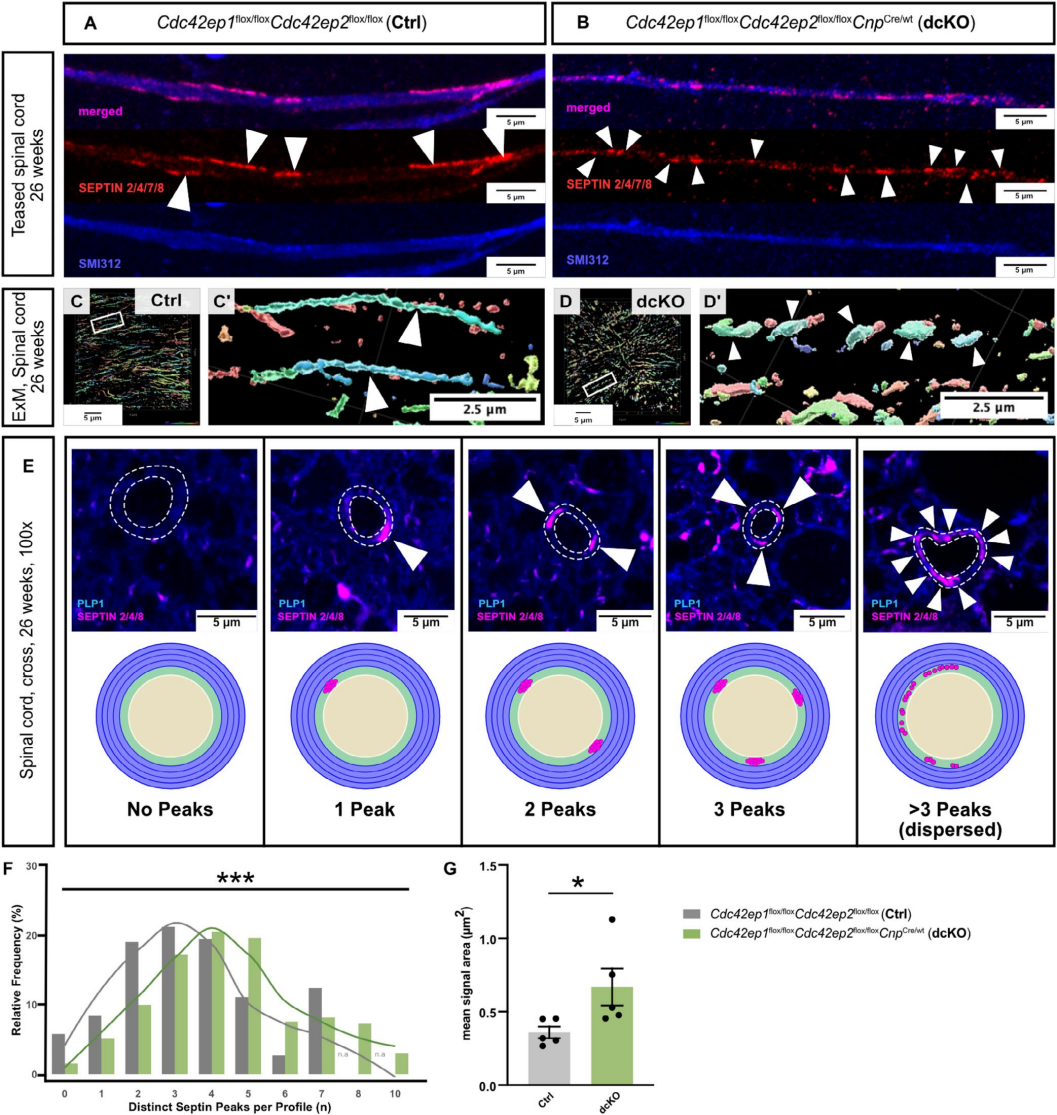
Disorganized higher order structure of myelin septin filaments when oligodendrocytes lack *Cdc42ep1* and *Cdc42ep2*. (A, B) Representative light micrographs of teased fibers dissected from dorsal spinal cords of *Cdc42ep1* ^*flox/flox*^; *Cdc42ep2* ^*flox/flox*^ (Ctrl, A) and *Cdc42ep1* ^*flox/flox*^; *Cdc42ep2* ^*flox/flox*^; *Cnp* ^*Cre/wt*^ (dcKO, B) mice at 26 weeks of age. All myelin septin subunits (SEPTIN2, SEPTIN4, SEPTIN7, and SEPTIN8) were collectively immunolabeled (shown in red) together with the axonal marker SMI312 (in blue) and imaged using a confocal microscope. Higher order septin structures of up to 12 μm length along the axon are readily detected in Ctrl (arrowheads in A), while septin‐immunopositive structures are more frequent but shorter and fragmented in dcKO mice (arrowheads in B). (C, D) Representative micrographs of selected myelin septins (SEPTIN2, SEPTIN4, and SEPTIN8) collectively immunolabeled on expanded spinal cord, imaged via multiphoton imaging, and 3‐dimensionally reconstructed. Objects are false‐colored in red, green, and blue according to their position along the z‐axis. Objects located in the same z‐plane are assigned the same color. (C, C′) Overview of myelin septin immunolabeling in expanded Ctrl spinal cord (C) and magnified view of the boxed region (C′). (D, D′) Overview of myelin septin immunolabeling in expanded dcKO spinal cord (D) and magnified view of the boxed region (D′). Note that higher order septin structures are readily detected in Ctrl (C′). In dcKO mice, septin‐immunopositive structures are more frequent but shorter and fragmented (D′). (E, G) Representative micrographs and schematics of myelin septins (SEPTIN2, SEPTIN4, SEPTIN8) collectively immunolabeled on cross‐sectioned spinal cord, superresolution imaging at 100× magnification (E), and genotype‐dependent quantification (F, G). Myelin septins are shown in magenta (arrows pointing at puncta in E), the marker for compact myelin PLP is in blue (outlined by stippled lines in E). (F) Genotype‐dependent quantification of the relative frequency of distinct septin puncta per axon/myelin‐profile indicate a shift towards a larger number of myelin septin puncta in control (gray) compared to dcKO (green) spinal cord. Ninety‐two axon/myelin profiles from five control mice and 100 axon/myelin profiles from five dcKO mice; number of myelin septin puncta by Chi‐squared test *p* = 6.151e−09. (G) Quantification of the mean signal area covered by septin puncta indicates that septin puncta are enlarged in dcKO mice. Mean ± SEM; datapoints represent individual mice; *n* = 5 mice per genotype; Students *t* test (*p* = 0.0482).

Considering that myelin septin filament subunits are merely reduced in abundance in dcKO myelin (Figure 6A–C) but not entirely lacking, we assessed the effect of CDC42EP1 and CDC42EP2 on myelin septin filaments by light microscopy. To this end, we dissected spinal cords of dcKO and Ctrl mice, prepared teased fibers following an established protocol (Jarjour and Sherman 2019), and collectively immunolabeled all myelin septin subunits (SEPTIN2, SEPTIN4, SEPTIN7, SEPTIN8), thereby visualizing myelin septins in single individualized ('teased') axon/myelin‐units. Septin‐immunopositive filament‐like structures of a length of up to ~12 μm along the axon were readily detected in teased spinal cords of Ctrl mice (Figure 7A). In contrast, teased spinal cords of dcKO mice displayed more but shorter, fragmented‐appearing septin‐immunopositive structures (Figure 7B).

To visualize myelin septin filaments in non‐teased tissue, we performed expansion microscopy. To this end, we dissected spinal cords of dcKO and Ctrl mice, expanded the tissue 6.7–7.7‐fold using an established protocol (Klimas et al. 2023), and collectively immunolabeled the myelin‐enriched septin subunits SEPTIN2, SEPTIN4, and SEPTIN8. Antibodies against SEPTIN7 were not included in this analysis because SEPTIN7 is part of all septin filaments in all cells (Spiliotis and Nakos 2021) and thus not well suited for specifically labeling myelin septins in the context of complex tissue. Septin‐immunopositive filament‐like structures of up to ~12 μm length were readily detected in expanded spinal cords of Ctrl mice (Figure 7C). Conversely, expanded spinal cords of dcKO mice displayed more but shorter, fragmented‐appearing septin‐immunopositive structures (Figure 7D).

To visualize myelin septins in non‐teased, non‐expanded tissue, we immunolabeled myelin‐enriched septin subunits SEPTIN2, SEPTIN4, and SEPTIN8 on cross‐sectioned spinal cords of Ctrl and dcKO mice. Super‐resolution microscopy allowed us to visualize septin puncta (also termed peaks) (Figure 7E). In Ctrl spinal cords, most cross‐sectioned axon/myelin‐units displayed zero, one, two, three, or four distinct septin peaks. In cross‐sectioned spinal cords of dcKO mice, however, we found a significant shift in the frequency distribution towards an increased number of septin peaks per axon/myelin‐unit (Figure 7F), in agreement with the presence of more but shorter, fragmented‐appearing septin‐immunopositive structures in dcKO myelin (Figure 7A–D). The area occupied by each individual septin peak was moderately but significantly increased (Figure 7H). Septin peaks were thus more dispersed in dcKO mice. Together, these data indicate that the higher order structure of myelin septin filaments is disorganized when CDC42EP1 and CDC42EP2 are missing.

## 3 Discussion

By assembling into sub‐membranous filaments, septins provide scaffolding and rigidity to the membranes they associate with (Gilden and Krummel 2010; Spiliotis and Gladfelter 2012; Tooley et al. 2009). In the adaxonal layer of CNS myelin, filaments composed of subunits SEPTIN2, SEPTIN4, SEPTIN7, and SEPTIN8 associate with the innermost compact myelin membrane, thereby preventing the formation of myelin outfoldings (Patzig et al. 2016). Myelin outfoldings also emerge in *Plp* ^*null/Y*^, *Mag* ^*null/null*^, *Cnp* ^*null/null*^, *Pten* ^*flox/flox*^; *Cnp* ^*Cre/WT*^, *Anln* ^*flox/flox*^; *Cnp* ^*Cre/WT*^, and *Pinch2* ^*flox/flox*^; *Cnp* ^*Cre/WT*^ mice, which is plausibly explained by the observed strong reduction of the abundance of myelin septins (Erwig et al. 2019b; Paes De Faria et al. 2022; Patzig et al. 2016). Normal brain aging also involves the emergence of myelin outfoldings (Hill et al. 2018; Peters 2002; Safaiyan et al. 2016; Yassa 2014), which at least in mice correlates with reduced abundance of myelin septins (Patzig et al. 2016). It is currently unknown how absence of myelin proteins as PLP, MAG, or CNP, or aging, may lead to reduced myelin septins. Possibly, interactions of particular septin monomers and the septin‐associated adaptor protein ANLN with the membrane lipid PI(4,5)P_2_ (Bertin et al. 2010; Liu et al. 2012; Zhang et al. 1999) are critical for the association of septins with the myelin membrane (Erwig et al. 2019b; Patzig et al. 2016). Remarkably, this recruitment mechanism appears conserved between the cleavage furrow of yeast (Bridges et al. 2014; Marquardt et al. 2021) and myelin, implying that myelin evolution included the recruitment of PI(4,5)P_2_‐ and ANLN‐dependent septin assembly from other cellular functions.

Following the hypothesis that other previously noted regulators of septins are also functionally relevant in myelin, we here tested the possibility that a family of CDC42‐effector proteins (CDC42EP) has a function in organizing septin multimers (Ageta‐Ishihara et al. 2015; Joberty et al. 2001; Schampera et al. 2025; Tomasso and Padrick 2023). Members of the CDC42EP family comprise multiple conserved sequence motifs including the C‐terminal ‘Borg homology domain’ (BH3) (Farrugia and Calvo 2016). The BH3 domain, at least of CDC42EP2 and CDC42EP5, is both necessary and sufficient for binding to septins, which facilitates filament bundling (Joberty et al. 2001). Mammals express five CDC42EP paralogs (Tomasso and Padrick 2023). We show here that CDC42EP1 and CDC42EP2 are expressed in oligodendrocytes and present in myelin. Their abundance in myelin depends on the presence of myelin septins, in agreement with the notion that members of the CDC42EP family are septin‐associated proteins (Tomasso and Padrick 2023). It is thus conceivable that, in myelin, CDC42EP1 and CDC42EP2 may be degraded if myelin septin filaments are lacking in *Septin8*‐mutants. On the other hand, the abundance of CDC42EP1, CDC42EP2, and septins in myelin is strongly reduced 10 months after recombination of the *Cdc42*‐gene in myelinating oligodendrocytes of *Cdc42* icKO mice, consistent with the notion that binding of at least some members of the CDC42EP family to septins is regulated by CDC42 (Ageta‐Ishihara et al. 2015; Joberty et al. 2001; Tomasso and Padrick 2023). Considering the key role of CDC42 in numerous cellular signaling pathways, it is not surprising that adult *Cdc42* icKO mice display complex myelin pathology. It is plausible that the observed myelin outfoldings are explained by the reduced abundance of septins in myelin when CDC42 is lacking, as previously shown for juvenile *Cdc42* cKO mice (Paes De Faria et al. 2022) and in the present analyses for tamoxifen‐induced adult *Cdc42* icKO mice. Oligodendroglial CDC42 is thus not only required for the development (Paes De Faria et al. 2022; Thurnherr et al. 2006) but also for the post‐developmental maintenance (this work) of normal myelin structure, which is facilitated by myelin septins as indicated by the tamoxifen‐induced deletion of the *Septin8* gene in adult oligodendrocytes (Patzig et al. 2016).

Upon targeting both the *Ccd42ep1* and *Ccd42ep2* genes, we found that single‐mutant mice lacking either CDC42EP1 or CDC42EP2 from oligodendrocytes are fully myelinated, while only CDC42EP1 cKO mice display myelin outfoldings as specific pathology. On the other hand, double‐mutant mice lacking oligodendroglial expression of both CDC42EP1 and CDC42EP2 displayed a considerably enhanced frequency of myelin outfoldings as compared to CDC42EP1 cKO mice, but also moderate other pathology. Thus, CDC42EP1 and CDC42EP2 serve overlapping functions in myelin sheath structure, mainly in preventing myelin outfoldings.

When assessing the CNS of mice lacking oligodendroglial expression of both CDC42EP1 and CDC42EP2, we found myelin outfoldings as a highly specific phenotype, in contrast to the complex pathology observed in *Cdc42* icKO mice or the previously analyzed *Cdc42* cKO mice (Thurnherr et al. 2006). It is thus likely that CDC42EP1 and CDC42EP2 are involved in only a subset or even only one of the functions of CDC42 in oligodendrocytes that is, the post‐transcriptional regulation of myelin septins. Considering that the levels of septin subunits in myelin are reduced but not entirely eliminated upon depletion of both CDC42EP1 and CDC42EP2, we considered it relevant to immunolabel septins and found that some septin subunits are still present in the adaxonal myelin compartment, and that smaller assemblies can form even when CDC42EP1 and CDC42EP2 are lacking. We interpret these findings to reflect that CDC42EP1/CDC42EP2 facilitate the polymerization of pre‐assembled hexamers into higher‐order structures, but probably not the oligomerization of monomeric subunits into hexamers. It is plausible that non‐oligomerized myelin septin subunits or small multimers may be degraded if not incorporated into a higher order structure myelin septin filament, a probable explanation for the reduced abundance of myelin septin subunits in dcKO myelin.

Myelin outfoldings or otherwise abnormal myelin sheaths are commonly quantified on electron micrographs of cross‐sectioned nerves (Erwig et al. 2019b; Iyer et al. 2024; Katanov et al. 2020; Snaidero and Simons 2017). However, the percentage of axon/myelin‐units displaying outfoldings as quantified in this way does not equal the actual percentage of affected myelin sheaths because myelin outfoldings do not span the entire length of a myelin sheath. Indeed, ultrathin sections have a thickness of ~60 nm and myelin outfoldings have lengths of ~1 μm up to 12 μm along the axon, according to reconstructions of focused ion beam‐scanning electron microscopy (FIB‐SEM) datasets of two models of spastic paraplegia (Steyer et al. 2022). However, depending on species and CNS region, the lengths of internodes as reported in the literature range from 50 μm up to 750 μm with medians of 200–250 μm (Ibrahim et al. 1995), from 27 μm up to 154 μm with a mean value of 82.7 ± 6.3 μm (Arancibia‐Cárcamo et al. 2017), or from ~30 μm up to > 200 μm with medians of 74.4 μm (Etxeberria et al. 2016). Each ultrathin section thus assesses just a part of a myelin sheath, which may comprise an outfolding at a different sectioning level. Therefore, the number of myelin sheaths with outfoldings is probably considerably higher than reflected by the percentage of affected axon/myelin profiles imaged per section. We estimate that ~4% of axon/myelin‐units with outfoldings per section in CDC42EP1/2 dcKO mice correspond to at least 24% of myelin sheaths with an outfolding, and possibly up to each sheath comprising at least one outfolding.

Myelin outfoldings and whorls are also a feature in the CNS of mice lacking neural Wiskott‐Aldrich syndrome protein (N‐WASP; also termed WASP‐like actin nucleation promoting factor, WASL) (Katanov et al. 2020) or actin‐related protein‐2/3 complex subunit 3 (ARPC3) (Zuchero et al. 2015) in myelinating cells. Interestingly, *N‐Wasp* ^*flox/flox*^; *Cnp* ^*Cre/WT*^ and *Arpc3* ^*flox/flox*^; *Cnp* ^*Cre/WT*^ mice also display a reduced percentage of myelinated axons and thinner myelin sheaths (Katanov et al. 2020; Zuchero et al. 2015). Thus, *N‐Wasp* ^*flox/flox*^; *Cnp* ^*Cre/WT*^ and *Arpc3* ^*flox/flox*^; *Cnp* ^*Cre/WT*^ mice show combined hyper‐ and hypomyelination (Katanov et al. 2020; Zuchero et al. 2015), in difference to *Septin8* ^*−/−*^, *Septin8* ^*flox/flox*^; *Cnp* ^*Cre/WT*^, and *Anln* ^*flox/flox*^; *Cnp* ^*Cre/WT*^ mice (Erwig et al. 2019b; Patzig et al. 2016), and the *Cdc42ep1* ^*flox/flox*^; *Cdc42ep2* ^*flox/flox*^; *Cnp* ^*Cre*^ dcKO mice investigated here, which all show myelin outfoldings, but no hypomyelination. N‐WASP is activated by CDC42 and in turn activates the actin‐related protein‐2/3 (ARP2/3) complex, which induces nucleation for actin filament assembly (Zuchero et al. 2015). In agreement with a function of N‐WASP in oligodendroglial process outgrowth (Bacon et al. 2007), actin depolymerization promotes myelin outfoldings (Zuchero et al. 2015). Thus, the N‐WASP‐ARP2/3‐actin pathway regulates both amount and morphology of myelin, while CDC42EP1/2 and septins specifically affect the latter via scaffolding. Considering that myelin septins have not been investigated in *N‐Wasp* ^*flox/flox*^; *Cnp* ^*Cre/WT*^ or *Arpc3* ^*flox/flox*^; *Cnp* ^*Cre/WT*^ mice, that the abundance of ARPC2 and ARPC3 is unchanged in *Cdc42ep1* ^*flox/flox*^; *Cdc42ep2* ^*flox/flox*^; *Cnp* ^*Cre*^ dcKO myelin, and that N‐WASP is not mass spectrometrically identified in CNS myelin, it remains currently speculative if, and at which level, the functions of N‐WASP‐ARP2/3‐actin and CDC42EP1/2‐septins in myelin morphology interconnect. We believe that, apart from the involvement of CDC42 as a master regulator, other interconnections may exist; however, their identification will be an interesting topic of future investigation.

### 3.1 Limitations of the Study

We have not succeeded in our aim to visualize the localization of CDC42EP1 and CDC42EP2 in myelin via fluorescence immunolabeling or cryo‐immuno electron microscopy (Tokuyasu method), despite testing multiple commercial and custom‐made antibodies and protocols. This is probably owed to their relatively low abundance, also when compared to that of CDC42, SEPTIN2, SEPTIN4, SEPTIN7, and SEPTIN8, which are readily detectable in myelin. The lack of direct visualization means that the presence of CDC42EP1 and CDC42EP2 in myelin can so far only be deduced from immunoblot analysis of biochemically purified myelin. Myelin fractions contain ≤ 5% contaminants from other cellular sources (Siems et al. 2025). We thus consider the strong genotype‐dependent changes in the abundance of CDC42EP1 and CDC42EP2 observed in myelin purified from the brains of *Septin8*‐deficient mice, *Cdc42* icKO mice, and *Cdc42ep1/Cdc42ep2* dcKO mice, as strong support for the notion that they are myelin constituents per se, and that their presence in myelin depends on CDC42 and myelin septins. However, it currently remains unknown whether CDC42EP1 and CDC42EP2 are localized in proximity to myelin septin filaments, which may reflect a physical association that could not be tested in the present study. The future development of more potent antibodies or nanobodies may thus help assessing the precise localization of CDC42EP1 and CDC42EP2 and their possible physical association with septins. Such tools may also enable explaining the observation that the abundance of CDC42 is strongly reduced in myelin of *Cdc42ep1/Cdc42ep2* dcKO but not of *Septin8*‐deficient mice. While one may have assumed a regulatory hierarchy from CDC42 via CDC42EP1/2 to SEPTIN2/4/7/8 filaments in myelin, our data indicate partial bidirectionality of interactions, which promises to be an interesting topic of future study.

### 3.2 Conclusion

Together, CDC42EP1 and CDC42EP2 regulate the higher order structure of myelin septins and thereby prevent myelin outfoldings. The reduced abundance of CDC42EP1 and CDC42EP2 in myelin lacking myelin septins indicates functional bi‐directionality. This supports the view that CDC42EP1 and CDC42EP2, as well as ANLN (Erwig et al. 2019b), can be considered septin‐associated proteins. When considering intercellular interactions in the CNS, it is of note that the presence of pathological myelin outfoldings per se does not induce astrocyte or microglial activation.

## 4 Methods

### 4.1 Mouse Lines

Mice harboring a floxed allele of the *Cdc42ep1* gene (also termed *Borg5*) were generated via site‐directed CRISPR/Cas9‐mediated genome editing, flanking exon 2 with loxP sites (allele name: *Cdc42ep1* ^*em1Bros*^). In brief, super‐ovulated C57BL/6N females were mated with C57BL/6N males, and fertilized zygotes were collected. CRISPR reagents (hCas9 mRNA, sgRNAs, pre‐assembled Cas9‐sgRNA ribonucleoprotein (RNP) complexes, and a double‐stranded DNA (dsDNA) template containing the two loxP‐site insertions, designed for homology‐directed repair (HDR)) were prepared and microinjected into the pronucleus and cytoplasm of pronuclear‐stage zygotes using a FemtoJet system (Eppendorf, Hamburg, Germany). CRISPR‐Cas9 components (sgRNAs, hCas9 mRNA) were delivered as RNA molecules rather than encoded in plasmid DNA because the transient nature of RNA‐based reagents reduces the risk of off‐target effects (Doench et al. 2016; Tycko et al. 2019). The sgRNAs targeting *Cdc42ep1* intron 1 (113–94 bp upstream of exon 2) and intron 2 (86–105 bp downstream of exon 2) were selected from the guide RNA candidates generated via the CRISPOR design tool (https://crispor.gi.ucsc.edu) version 4.7 (Concordet and Haeussler 2018; Haeussler et al. 2016). Correct site‐specific integration of the two loxP‐sites into the intronic regions flanking exon 2 was verified by localization PCR using primers flanking the homology arms of the HDR template. The resulting PCR products were validated by Sanger sequencing. To further validate the presence and sequence integrity of the inserted loxP‐sites, loxP‐containing PCR products were cloned, and individual clones were sequenced. A scheme of the locus, the sequence of the HDR fragment, and the sgRNA sequences are provided in Figure S3. Zygotes were transferred to C57Bl6/N females. To recombine the *Cdc42ep1* ^*flox*^ allele in oligodendrocytes, we interbred *Cdc42ep1* ^*flox*^ mice with a *Cnp* ^*Cre/wt*^ driver line (Lappe‐Siefke et al. 2003), gaining *Cdc42ep1* ^*flox/flox*^; *Cnp1* ^*Cre/wt*^ mice (also termed EP1 cKO). Littermate mice with the genotype *Cdc42ep1* ^*flox/flox*^ served as controls and were designated as Ctrl.

To generate mice harboring a floxed allele of the *Cdc42ep2* gene (also termed *Borg1*), *Cdc42ep2* ^*tm1a(EUCOMM)Hmgu*^ embryonic stem (ES) cells were obtained from the European Conditional Mutagenesis Program (Eucomm, Munich, Germany). ES were microinjected into blastocysts derived from FVB mice, and embryos were transferred to pseudo‐pregnant foster mothers. Chimeric male offspring were first crossed with wild‐type C57BL/6N mice to produce F1 offspring. Mice carrying the *Cdc42ep2* ^*lacZ/neo*^ allele, which includes the full targeting cassette (http://www.mousephenotype.org/data/genes/MGI:1929744), were identified by genotyping PCR (see below). The lacZ/neo cassette was excised in vivo upon interbreeding with mice expressing FLIP recombinase (129S4/SvJae‐Sor‐Gt(ROSA)26Sortm1(FLP1)Dym/J; backcrossed into C57BL/6N), yielding *Cdc42ep2* ^*flox*^ mice. To recombine the *Cdc42ep2* ^*flox*^ allele in oligodendrocytes, *Cdc42ep2* ^*flox*^ mice were interbred with a *Cnp* ^*Cre/wt*^ driver line (Lappe‐Siefke et al. 2003), gaining *Cdc42ep2* ^*flox/flox*^; *Cnp* ^*Cre/wt*^ mice (also termed EP2 cKO). Littermate mice with the genotype *Cdc42ep2* ^*flox/flox*^ served as controls and were designated as Ctrl.

To recombine both *Cdc42ep1* and *Cdc42ep2* in oligodendrocytes, mice were interbred yielding *Cdc42ep1* ^*flox/flox*^; *Cdc42ep2* ^*flox/flox*^; *Cnp* ^*Cre/wt*^ mice (also termed dcKO). Genotypes were determined by genomic PCR. Primers to determine the *Cdc42ep1* ^*flox*^ allele were 5′‐GCATTCCCTCATTCGGTCACAC (internal primer #38247), 5′‐TGACTTTACTAAGGCGCTGTCC (#38248), 5′‐GCCACTCTGGTTACGGTGAGGA (#38249), and 5′‐CACCATGCCCCGGCTGACTTC (#38250). Primers to determine *Cdc42ep2* ^*flox*^ allele were 5′‐CATCTCTCCAGCCCTCAAGTG, 5′‐GTGCGTTTGTGAGTGTGGGC, and 5′‐GGAGAGCTGGAGGCAGAAAGG. Primers to determine the *Cnp* ^*Cre*^ allele were 5′‐GCCTTCAAACTGTCCATCTC, 5′‐CAGGGTGTTATAAGCAATCCC, 5′‐CCCAGCCCTTTTATTACCAC, and 5′‐CCTGGAAAATGCTTCTGTCCG.

EP1 cKO, EP2 cKO, dcKO, and respective control mice were bred and raised at the MPI‐NAT (Göttingen, Germany) with 2–5 mice per cage. Mice had access to food and water *ad libitum* and experienced a 12‐h light–dark cycle. All experiments were in accordance with the German Animal Welfare Law (Tierschutzgesetz der Bundesrepublik Deutschland, TierSchG). For procedures of sacrificing rodents for subsequent preparation of tissue, all regulations given in TierSchG 4 were followed. Since the sacrificing of mice is not an experiment on animals according to TierSchG 7 Abs. 2 Satz 3, no specific authorization was required. The animal facility at the MPI‐NAT is registered at the Niedersächsisches Landesamt für Verbraucherschutz und Lebensmittelsicherheit (LAVES) according to TierSchG 11 Abs. 1. According to TierSchG and the regulation about animals used in experiments dated 11th August 2021 (Tierschutz‐Versuchstierverordnung, TierSchVersV), an animal welfare officer and an animal welfare committee are established for the institute.

To recombine the previously generated *Cdc42* ^*flox*^ allele (Wu et al. 2006) in oligodendrocytes of adult mice, *Cdc42* ^*flox/flox*^ mice were interbred with mice expressing tamoxifen‐inducible Cre recombinase under control of the *Plp* promoter (*Plp* ^*CreERT2*^) (Leone et al. 2003), gaining *Cdc42* ^*flox/flox*^; *Plp* ^*CreERT2*^ mice (also termed Cdc42 icKO) and *Cdc42* ^*flox/flox*^ littermate controls. Cdc42 icKO and respective Ctrl mice were bred and raised at the i3S (Porto, Portugal). Primers to determine the *Cdc42* ^*flox*^ allele were 5′‐TCTGCCATCTACACATACAC and 5′‐ATGTAGTGTCTGTCCATTGG. Primers to detect the *Plp* ^*CreERT2*^ allele were 5′‐ACCAGGTTCGTTCACTCATGG and 5′‐AGGCTAAGTGCCTTCTCTACA. Control and experimental animals were housed under a 12‐h light/dark cycle with *ad libitum* access to water and standard chow. All housing, breeding, and handling procedures adhered strictly to established protocols. Animal experiments were conducted in accordance with approvals from the local Animal Ethics Committee, the Portuguese Veterinary Authority (DGAV), and relevant EU directives (Project Licenses DGAV 11770/2014 and 002803/2021). Mice were injected intraperitoneally daily with freshly prepared 2 mg tamoxifen per day (Sigma T5648, 20 mg/mL in 9:1 sunflower oil: ethanol mixture) for five consecutive days starting at postnatal day 21.

### 4.2 Biochemical Purification of CNS Myelin

Myelin purification was performed as previously described using sucrose gradient centrifugation and osmotic shocks (Erwig et al. 2019a). Half brains of mice were dissected after sacrificing by cervical dislocation and homogenized in 5 mL 0.32 M sucrose containing protease inhibitor (Roche Complete, Roche Diagnostics GmbH, Mannheim, Germany) using a T10 ULTRA‐TURRAX homogenizer (IKA, Staufen, Germany). Purified myelin samples were measured for protein concentration based on the Lowry assay (Lowry et al. 1951) by using the DC Protein Assay Kit (Bio‐Rad, Hercules, USA) and the manufacturer's microplate assay protocol. Optical density was measured at 650 nm with an Eon High Performance Microplate Spectrophotometer (BioTek, Vermont, USA) with Gen5 software (BioTek Instruments, Bad Friedrichshall, Germany).

### 4.3 Myelin Proteome Analysis

In‐solution digestion of myelin proteins by filter‐aided sample preparation (FASP) (Erwig et al. 2019a) and LC–MS analysis by different MS^E^‐type data‐independent acquisition (DIA) mass spectrometry approaches was performed as recently established for mouse PNS and CNS myelin (Jahn et al. 2020; Siems et al. 2020). Briefly, protein fractions corresponding to 10 μg myelin protein were dissolved in lysis buffer (1% ASB‐14, 7 M urea, 2 M thiourea, 10 mM DTT, 0.1 M Tris pH 8.5) and processed according to a CHAPS‐based FASP protocol in centrifugal filter units (30 kDa MWCO, Merck Millipore). After removal of the detergents, protein alkylation with iodoacetamide, and buffer exchange to digestion buffer (50 mM ammonium bicarbonate (ABC), 10% acetonitrile), proteins were digested overnight at 37°C with 400 ng trypsin. Tryptic peptides were recovered by centrifugation and extracted with 40 μL of 50 mM ABC and 40 μL of 1% trifluoroacetic acid (TFA), respectively. For quantification according to the TOP3 approach, combined flow‐throughs were spiked with 10 fmol/μl of Hi3 *E. coli* standard (Waters Corporation; contains a set of quantified synthetic peptides derived from *E. coli* chaperone protein ClpB) and directly subjected to LC–MS analysis.

Tryptic peptides were separated by nanoscale reversed‐phase UPLC and mass spectrometrically analyzed on a quadrupole time‐of‐flight mass spectrometer with ion mobility option (Synapt G2‐S/‐Si, Waters Corporation) as recently described in detail (Jahn et al. 2020; Siems et al. 2020). The samples were first analyzed in the ion mobility‐enhanced DIA mode with drift time‐specific collision energies referred to as UDMS^E^ (providing increased proteome coverage at the cost of dynamic range for the accurate quantification of medium‐ to low‐abundant proteins) and rerun in a data acquisition mode without ion mobility separation of peptides referred to as MS^E^ (providing increased dynamic range at the cost of proteome coverage for the accurate quantification of exceptionally abundant myelin proteins) (see (Siems et al. 2020) for details). Continuum LC–MS data were processed using Waters ProteinLynx Global Server (PLGS) and searched against a custom database compiled by adding the sequence information for *E. coli* chaperone protein ClpB and porcine trypsin to the UniProtKB/Swiss‐Prot mouse proteome (CDC42EP: release 2021‐04, 17,090 entries; CDC42: release 2022‐05, 17,137 entries) and by appending the reversed sequence of each entry to enable the determination of false discovery rate (FDR) set to 1% threshold.

For post‐identification analysis including TOP3 quantification of proteins, the freely available software ISOQuant (Distler et al. 2014) was used as described (Jahn et al. 2020; Siems et al. 2020). Only proteins represented by at least two peptides (minimum length six amino acids, score ≥ 5.5, identified in at least two runs) were quantified as parts per million (PPM) that is, the relative amount (w/w) of each protein in respect to the sum over all detected proteins. FDR for both peptides and proteins was set to a 1% threshold and at least one unique peptide was required. Proteins identified as contaminants from blood (albumin, hemoglobin) or skin/hair cells (keratins) were removed and potential outlier proteins were revised by inspecting the quality of peptide identification, quantification and distribution between protein isoforms. Filtered protein lists were subjected to statistical analysis with the Bioconductor R packages ‘limma’ and ‘q‐value’ to detect significant changes in protein abundance by moderated t‐statistics as described (Jahn et al. 2020; Siems et al. 2020). Proteome profiling of control and mutant myelin was performed with three biological replicates per condition and duplicate digestion as a technical replicate, resulting in a total of 24 (CDC42EP) or 12 (CDC42) LC–MS runs to be compared. For data visualization by volcano plots, *q* values were plotted against fold‐change after −log10 and log2 transformation, respectively.

### 4.4 Immunoblotting

Immunoblotting was performed as described (Gargareta et al. 2022). Samples were diluted in 1× SDS sample buffer (40% Glycerol, 240 mM Tris/HCl pH 6.8, 8% SDS, 0.04% Bromphenol blue) and 5% Dithiotheitol (DTT). Proteins were separated using acrylamide SDS gels of varying concentrations of acrylamide 10%, 12% or 15%. Samples were incubated for 10 min at 40°C prior to loading in the SDS Gel. 5 μL of a molecular marker (PageRuler️ Plus Prestained Protein Ladder, Thermo Fisher Scientific, St. Leon‐Rot, Germany) was added to each gel to facilitate the detection of the molecular weight of the proteins of interest and between 1 and 20 μg of sample was loaded after short vortexing into each pocket of the gel. Proteins were separated in the gel by applying 180 V of constant current for about 1 h with a power‐supply system (Bio‐Rad, Hercules, USA) in a Novex Semi‐Dry Blotter (Invitrogen, Karlsruhe, Germany). Proteins were transferred to activated polyvinylidene difluoride (PVDF) membranes (GE Healthcare Life Science, Chicago, USA). Transfer was controlled via incubating membranes 5 min in 1× Fast Green solution (0.005 mg/mL Fast Green (Merck KGaA, Darmstadt, Germany), 30% Methanol, 6.7% Acetic acid, 63.3% ddH_2_O), followed by two short washing steps with Fast Green washing solution. The fluorescent signal from the Fast Green staining was detected at 670 nm using a ChemoStar imaging system (INTAS Science Imaging Instruments GmbH, Göttingen, Germany). Membranes were then washed in TBS‐T (50 mM Tris/HCl, pH 7.5, 150 mM NaCl, 0.05% Tween‐20) and subsequently blocked in blocking buffer consisting of 5% (w/v) non‐fat dry milk (Frema instant skimmed milk powder) in 1× TBS‐T for 1 h at room temperature. Primary antibodies were also diluted in blocking buffer, and the membranes incubated in these solutions for 1–4 nights at 4°C. The membranes were washed four times with TBS‐T at room temperature before incubating the membrane in horseradish‐peroxidase (HRP‐) coupled secondary antibody solutions (1:10,000), diluted in blocking solution for 1 h at room temperature. After incubation with secondary antibodies, the membranes were washed several times in TBS‐T (4×; 5 min) and developed using enhanced chemiluminescent detection (ECL) in 1:1 ratio (Western LightningR Plus‐ECL or SuperSignal West Femto Maximum Sensitive Substrate; Thermo Fischer Scientific, St. Leon‐Rot, Germany) and imaged in a chemiluminescent imager (INTAS Science Imaging Instruments GmbH, Göttingen, Germany).

Primary antibodies were specific for CDC42 (Santa Cruz, 1:500, #sc‐8401), CDC42EP1 (custom‐made rabbit polyclonal antibody against mouse CDC42EP1 epitope VEKHSNRDRDRDPDH, Pineda, 1:1000), CDC42EP2 (Proteintech Group, 1:300, #11824‐1‐AP), SEPTIN2 (Proteintech Group, 1:500, #11397‐1‐AP), SEPTIN4 (IBL, 1:200, #JP18987), SEPTIN7 (IBL, 1:5000, #18991), SEPTIN8 (Proteintech Group, 1:500, #11769‐1‐AP), or ATP1a3 (Abcam, 1:1000, #ab182571). Secondary HRP‐coupled antibodies were HRP‐goat‐anti‐mouse IgG (Dianova), IgG (Dianova, #115‐03‐003) (Dianova, Hamburg, Germany) or HRP‐goat anti‐rabbit IgG (Dianova, #111‐035‐003).

### 4.5 Quantitative Real Time PCR

RNA isolation was performed on corpora callosa dissected from mice sacrificed by cervical dislocation at age 20 weeks. The tissue was homogenized in Trizol (Qiagen, Hilden, Germany) with Teflon beads in a Precellys 24 tissue homogenizer system (2×; 30 s) (Bertin Technologies, Montigny‐le‐Bretonneux, France), and RNA was extracted using the Miniprep kit (Qiagen, Hilden, Germany). The quality and concentration of the eluted RNA precipitate were measured using the NanoDrop 2000 spectrophotometer (Thermo Fisher Scientific, St. Leon‐Rot, Germany). The concentration of the isolated RNA was adjusted to 300 ng/μL for each sample. Single‐strand cDNA was synthesized by using SuperScriptIII reverse transcriptase (200 U/μL) (Invitrogen, Karlsruhe, Germany) and diluted to 5 ng/μL. Quantitative real‐time PCR was performed according to the manufacturer's protocol using SYBR Green Master Mix (Promega, Fitchburg, USA). Primers for genes were custom‐made (Department of Molecular Neurobiology, MPI‐NAT, Göttingen, Germany). Reactions were performed in triplicates; for each triplicate, 10 ng of cDNA were used. PCR reactions were carried out in 384 well plates in a LightCycler 480 II (Roche Diagnostics GmbH, Mannheim, Germany). The mRNA abundance was quantified using the LightCycler 480 Instrument II software and normalized to the mean of the standards *Hprt* and *Rplp0*. For each sample, ΔΔCT values were calculated, and statistical analysis was performed in GraphPad Prism 10 (Dotmatics, Boston, USA).

Primers were specific for *Anln* (5′‐ACAATCCAAGGACAAACTTGC, 5′‐GCGTTCCAGGAAAGGCTTA), *Aspa* (5′‐AGTGGAGACATGGCTGCTGTT, 5′‐GATCTCCAGGGTGCAATGGT), *Cdc42* (5′‐CGACCGCTAAGTTATCCACAG, 5′‐GAGTTATCTCAGGCACCCACTT), *Cdc42ep1* (5′‐TCCGCCTCCCATCTCGCCCAT, 5′‐CAGACTCCAGGCCGTAACC), *Cdc42ep2* (5′‐TGGTCCTCTATCCTAGCCGCA, 5′‐TAGTCCAGAGTTGAGAAGCTTGAG), *Gfap* (5′‐GACAGAGGAGTGGTATCGGTC, 5′‐AGTCGTTAGCTTCGTGCTTGG), *Hprt* (5′‐TCCTCCTCAGACCGCTTTT, 5′‐CCTGGTTCATCATCGCTAATC), *Iba1* (5′‐CAAGAGCCTGGATAGGAGACC, 5′‐CATCAGCTTCTGCTGACACC), *Lamp2* (5′‐AAGGTGCAACCTTTTAATGTGAC, 5′‐TGTCATCATCCAGCGAACAC), *Mog* (5′‐ATGAAGGAGGCTACACCTGC, 5′‐CAAGTGCGATGAGAGTCAGC), *Rplp0* (5′‐GATGCCCAGGGAAGACAG, 5′‐ACAATGAAGCATTTTGGATAATCA), *Septin2* (5′‐TCCTGACTGATCTCTACCCAGAA, 5′‐TCCTGACTGATCTCTACCCAGAA), *Septin4* (5′‐ACTGACTTGTACCGGGATCG, 5′‐TCTCCACGGTTTGCATGAT), *Septin7* (5′‐AGAGGAAGGCAGTATCCTTGG, 5′‐TTTCAAGTCCTGCATATGTGTTC), *Septin8* (5′‐CCTCTGAGGAAAGACAAGGACA, 5′‐GCTCCATCAAGGAATAGTGACA).

### 4.6 Re‐Analysis of scRNA‐Seq Data

The publicly available scRNA‐seq dataset GSE77533 (Marques et al. 2016) was analyzed for transcript expression. Data preprocessing followed Seurat v3.1.4 (Gargareta et al. 2022), including LogNormalize normalization, selection of the 2000 most variable features, and scaling. All six clusters annotated as myelinating oligodendrocytes (MOL) according to (Marques et al. 2016) were subset, and DGE between *Cdc42* and *Cdc42ep1/2/3/4/5* transcripts was performed using Seurat v4.3.2 with the FindMarkers function (Wilcoxon rank‐sum test; min.pct = 0.1; adjusted *p* < 0.05; minimum average log2FC ±0.25). Violin plots were generated from the scaled data using ggplot2. *N* = 998 cells.

### 4.7 Transmission Electron Microscopy

Optic nerves or spinal cords were dissected for analysis after mice were sacrificed by cervical dislocation. The tissue was collected and directly fixed for electron microscopy analysis (4% PFA, 2.5% glutaraldehyde in 0.1 M phosphate buffer (PB) (0.36 g NaH_2_PO_4_ × H_2_O, 3.1 g Na_2_HPO_4_ × 2H_2_O in 200 mL 1% NaCl)). The samples were embedded in EPON following a protocol of osmification, dehydration and Epon infiltration as described (Weil et al. 2019). The samples were placed in silicone molds which were filled with Epon and labeled before polymerization for 24 h at 60°C. Once the Epon resin blocks hardened, the blocks were sectioned using an ultramicrotome (UC7, Leica Microsystems, Wetzlar, Germany) with a diamond knife (Ultra 35°, 3.0 mm, Diatome AG, Switzerland). Ultra‐thin sections (60 nm) were collected and transferred into copper grids (Science Services, Munich, Germany), which were coated with formvar. The contrast was enhanced by applying Uranyless (Electron Microscopy Services, Hatfield, USA) for 30 min followed by five washing steps with ddH_2_O and drying. Images were acquired with two different transmission electron microscopes: LEO EM912 Omega (Zeiss, Oberkochen, Germany) and EM900 (Zeiss, Oberkochen, Germany). Optic nerves were imaged at a magnification of 7000×. Per mouse 10–15 non‐overlapping images were acquired and analyzed using FIJI (Schindelin et al. 2012). For g‐ratio analysis a grid was overlaid and only axons positioned at grid‐line intersections were included in the measurement. The Feret diameter of the axon and of the corresponding myelin sheath was measured and the ratio (g‐ratio) between the two was calculated. In cases where the inner tongue was visible it was also measured and the g‐ratio calculation adapted to correct for this. For the analysis of myelin characteristics all visible axonal profiles were labeled into one of the following categories: normal appearing axon/myelin unit, non‐myelinated axon, myelin outfolding, myelin whorl and other pathology.

### 4.8 Immunohistochemistry

Mice were injected intraperitoneally with 0.2 mg Avertin per 10 g body weight ensuring lethal overdose prior to transcardial perfusion with Hank's buffered salt solution (HBSS, Gibco, Thermo Fisher Scientific) followed by 4% Paraformaldehyde (PFA) in 0.1 M phosphate buffer for tissue fixation. Dissected tissue was post‐fixed in 4% PFA in 0.1 M phosphate buffer. Samples were embedded in paraffin using an automated embedding system (Leica Microsystems, Wetzlar, Germany) and sectioned into 5 μm sections with a microtome (HM400R, MICROM, Thermo Fischer Scientific). For chromogen labelling, primary antibodies were specific for aspartoacylase (ASPA) (US Biological, 1:4000, #MBS422542), glial fibrillary acidic protein (GFAP) (Novo Castra, 1:200, #NCL‐L‐GFAP‐GA5), lysosome‐associated membrane glycoprotein 2 (LAMP2/MAC3) (PD Pharmingen, 1:400, # 770064), ionized calcium‐binding adapter molecule 1 (IBA1) (abcam, 1:1000, #ab5076), or amyloid beta precursor protein (APP) (Chemicon, 1:1000, #MAB348). IBA1 and ASPA antibodies were detected using the Vector Elite ABC kit (Vector Labs, Newark, Germany); GFAP and APP antibodies were detected using the LSAB2 kit (Dako, Glostrup, Denmark). Sections were imaged at 40× magnification with a AxioImager Z1 bright‐field light microscope system (Zeiss, Oberkochen, Germany) coupled to an AxioCam MRc Camera (Zeiss, Oberkochen, Germany) and controlled by Zeiss Zen 1.0 software (Zeiss, Oberkochen, Germany). Analysis of chromogenic neuropathology labelling was performed as described (de Monasterio‐Schrader et al. 2013; Lüders et al. 2017) using FIJI and an ImageJ plugin to analyze the immune‐positive staining area for IBA1‐ or GFAP‐ or MAC3/LAMP2‐immunolabeling. For the analysis of APP‐ or ASPA‐positive cells/structures were manually counted and normalized to the assessed area.

For fluorescent immunolabeling of paraffin sections, slides were first deparaffinized and rehydrated. Antigen retrieval was performed using basic Tris/EDTA buffer (pH 9.0), and slides were boiled 10 min at 600 W in a regular microwave oven (Panasonic, Wiesbaden, Germany). After cooling down for about 20 min, slides were rinsed 1 min with ddH_2_O. For permeabilization, slides were treated 15 min with 0.1% Triton X‐100 in 1× PBS and subsequently washed briefly in 1× PBS. To prevent unspecific binding of antibodies, blocking buffer (10% goat serum in 1 PBS) was applied 1 h at room temperature. Primary antibodies were diluted in blocking buffer and incubated overnight at 4°C. Primary antibodies for fluorescent staining were specific for CDC42 (Santa Cruz, 1:100, #sc‐8401), CDC42EP1 (Custom Pineda, 1:100), CASPR1 (abcam, 1:500, #ab34151), PLP1 (clone aa3, Custom, 1:400), SEPTIN2 (Proteintech Group, 1:500, #11397‐1‐AP), SEPTIN4 (IBL, 1:200, #JP18987), SEPTIN7 (Proteintech Group, 1:100, #66542‐1‐Ig), SEPTIN7 (IBL, 1:500, #18991), or SEPTIN8 (Proteintech Group, 1:500, #11769‐1‐AP). The following day, slides were washed 3 × 5 min in 1× PBS, and subsequently diluted (1:500) fluorescent secondary antibodies (α‐rabbit STAR‐RED, α‐mouse STAR‐ORANGE, α‐rat STAR‐ORANGE, α‐rat STAR‐GREEN 460 L, abberior Instruments GmbH, Göttingen, Germany) were applied for 1–2 h at room temperature together with DAPI (1:10,000) to label cell nuclei. Finally, slides were washed 3 × 5 min in 1× PBS before mounting in embedding medium ProLong Glass (Invitrogen, Karlsruhe, Germany) and storing at 4°C until imaging. Images were acquired with a STEDYCON system (abberior Instruments GmbH, Göttingen, Germany) at 100× magnification.

Teased spinal cord fibers were prepared as described (Jarjour and Sherman 2019). Mice were injected intraperitoneally with 0.2 mg Avertin per 10 g body weight ensuring lethal overdose prior to perfusion with HBSS and subsequent immersion fixation in 4% PFA. Teased fiber slides were briefly washed in 1× PBS. For permeabilization, slides were washed with MeOH for 5 min. Slides were then incubated in blocking buffer (3% [v/v] Normal donkey serum (NDS), 2% [w/v] bovine serum albumin (BSA), 0.1% [v/v] Triton X‐100 in 1× PBS) for 1 h at room temperature on a shaker. The primary antibodies were diluted in blocking buffer and applied to the slides overnight at 4°C. Primary antibodies were SMI312 (Biolegend, 1:500, #837904), SEPTIN2 (Proteintech Group, 1:500, #11397–1‐AP), SEPTIN4 (IBL, 1:500, #JP18987), SEPTIN7 (IBL, 1:5000, #18991), SEPTIN8 (Proteintech Group, 1:500, #11769‐1‐AP). The next day, slides were washed three times in blocking solution, 10 min each either at room temperature. Fluorescent secondary antibodies were diluted in blocking buffer (1:500) and applied to the slides for 1 h at 4°C (α‐rabbit STAR‐RED, α‐mouse STAR‐ORANGE, abberior Instruments GmbH, Göttingen, Germany). Slides were washed three times in blocking solution for 10 min per step and afterwards two times in 1× PBS (10 min each). Excess PBS was removed and slides were mounted using ProLong Glass (Invitrogen, Karlsruhe, Germany). Images were acquired with a LSM880 with Airyscan (Carl Zeiss Microscopy GmbH, Jena, Germany) using a 40× oil immersion objective with 1.4 NA.

### 4.9 Expansion Microscopy (ExM)

Mice were injected intraperitoneally with 0.2 mg Avertin per 10 g body weight ensuring lethal overdose prior to the perfusion with 4% PFA and subsequent dissection. The spinal cord column was dissected and post‐fixed in 10% PFA and 30% sucrose. For processing, spinal cords were embedded in 10% gelatine prior to vibratome (VT1200S, Leica Microsystems, Wetzlar, Germany) sectioning. Longitudinal sections were cut to 100 μm thickness at 0.6 m/s speed in ice cold 1× PBS. Spinal cords were expanded according to the adapted “Magnify” protocol (Klimas et al. 2023), a universally applicable method of expanding various types of tissue or cell samples. Briefly, by embedding the sample in a specific hydrogel and treatment with water, the gels expand isotropically in all orientations while retaining proteins and lipids without additional anchoring steps. Fluorescence labelling was applied post‐gelation to visualize the septin filaments at higher resolution and samples expanded to dimensions between 6.7 and 7.7 times. A LaVision TriM Scope II Multiphoton Microscope (LaVision BioTec GmbH, Bielefeld, Germany) equipped with a tunable CRONUS 2P femtosecond laser (Light Conversion, Vilnius, Lithuania) and a Nikon ELWD ADM 40× (0.6 NA) objective with a working distance up to 3.6 mm was used to acquire 300 μm z‐stacks within the respective hydrogel‐embedded sample. A Difference of Gaussian filter was applied to the resulting image stacks with the CLIJ2 (Haase et al. 2020) plugin in FIJI (Schindelin et al. 2012). The expansion factor was taken into account when establishing the final pixel dimension of the respective expanded sample. Imaris 10.2.0 (Bitplane AG, Schlieren, Switzerland) was used for further visualization and processing. Primary antibodies specific for SMI312 (Biolegend, 1:200, #837904), SEPTIN2 (Proteintech Group, 1:200, #11397‐1‐AP), SEPTIN4 (IBL, 1:200, #JP18987) and SEPTIN8 (Proteintech Group, 1:200, #11769‐1‐AP) were used. Fluorescent secondary antibodies were anti‐mouse Alexa488 (Invitrogen, 1:500, #A‐11001) and anti‐rabbit Alexa568 (Invitrogen, 1:500, #A‐11011).

### 4.10 Quantifications and Statistical Analysis

Data was assessed statistically using GraphPad Prism 10 (Dotmatics, Boston, USA) or RStudio (Version 2024.09) and whenever possible, experiments were analyzed in a blinded manner. Firstly, data was assessed for normality by Shapiro–Wilk test. If two groups were compared and both were parametrically distributed, unpaired Students t‐test was performed. For non‐parametric data, Mann–Whitney test was chosen if two groups were compared. For comparison of more than two groups, one‐way ANOVA with Tukey's post hoc test was applied. Multiple t‐tests were subjected to Holm‐Šidák corrections. Frequency distributions were assessed using Chi‐squared tests. Levels of significance are indicated as n.s = not significant, *p < 0.05, **p < 0.01, ***p < 0.001 with exact *p* values indicated in the figure legends. Calculations and illustrations of data were performed using FIJI (Schindelin et al. 2012), GraphPad Prism 10 (Dotmatics, Boston, USA) and Affinity Designer 2 (Serif Europe Ltd., Nottingham, UK).

## Author Contributions

**Sophie Hümmert:** investigation, data analysis, writing‐original draft, writing‐review and editing. **Joana Paes de Faria:** unpublished materials, methodology, writing‐review and editing. **Olaf Jahn:** methodology, data analysis, writing‐review and editing. **Ege Bilgin:** investigation, Writing‐review and editing. **Nikola Łukasik:** investigation, writing‐review and editing. **Chethana Rao:** methodology, writing‐review and editing. **Mišo Mitkovski:** conceptualization and methodology of stedycon microscopy and image analysis, writing‐review and editing. **Fritz Benseler:** methodology, writing‐review and editing. **Nils Brose:** supervision, writing‐review and editing. **Sophie B. Siems:** conceptualization, writing‐review and editing. **Sandra Goebbels:** unpublished materials, writing‐review and editing. **Wiebke Möbius:** methodology, supervision, Writing – review and editing. **Helge Ewers:** methodology, supervision, writing – review and editing. **João B. Relvas:** unpublished materials, conceptualization, supervision, writing‐review and editing. **Hauke B. Werner:** conceptualization, funding, supervision, writing – original draft, writing – review and editing.

## Funding

This work was supported by Deutsche Forschungsgemeinschaft (2720/5‐1) to H.B.W.

## Ethics Statement

*Cdc42ep1* ^*flox/flox*^; *Cdc42ep2* ^*flox/flox*^; *Cnp* ^*Cre/wt*^ and respective single‐mutant and control mice were bred and raised at the MPI‐NAT (Göttingen, Germany) with 2–5 mice per cage. Mice had access to food and water ad libitum and experienced a 12‐h light–dark cycle. All experiments were in accordance with the German Animal Welfare Law (Tierschutzgesetz der Bundesrepublik Deutschland, TierSchG). For procedures of sacrificing rodents for subsequent preparation of tissue, all regulations given in TierSchG 4 were followed. Since sacrificing of mice is not an experiment on animals according to TierSchG 7 Abs. 2 Satz 3, no specific authorization was required. The animal facility at the MPI‐NAT is registered at the Niedersächsisches Landesamt für Verbraucherschutz und Lebensmittelsicherheit (LAVES) according to TierSchG 11 Abs. 1. According to TierSchG and the regulation about animals used in experiments dated 11th August 2021 (Tierschutz‐Versuchstierverordnung, TierSchVersV), an animal welfare officer and an animal welfare committee are established for the institute. *Cdc42* ^*flox/flox*^; *Plp* ^*CreERT2*^ and control mice were bred and raised at the i3S (Porto, Portugal) and housed under a 12‐h light/dark cycle with ad libitum access to water and standard chow. All procedures were in accordance with approvals from the local Animal Ethics Committee, the Portuguese Veterinary Authority (DGAV), and relevant EU directives (Project Licenses DGAV 11770/2014 and 002803/2021).

## Conflicts of Interest

The authors declare no conflicts of interest.

## Supporting information


**Figure S1:** Oligodendrocytes express *Cdc42, Cdc42ep1*, and *Cdc42ep2* according to bulk RNA‐Seq data of immunopanned cells.
RNA‐Seq of cells immunopanned from mouse cortices shows that *Cdc42*, *Ccd42ep1*, and *Ccd42ep2* transcripts are detected in both newly formed oligodendrocytes and myelinating oligodendrocytes. Re‐analysis of data from (Zhang et al. 2014). Mean +/‐SEM; datapoints represent individual experiments. FPKM, fragments per kilobase of transcript per million fragments mapped.
**Figure S2:** Myelin outfoldings in the spinal cord when oligodendrocytes lack Cdc42 or both Cdc42ep1 and Cdc42ep2.
A‐D Representative electron micrographs of cross‐sectioned spinal cords show that myelin outfoldings are the main pathology in spinal cords of *Cdc42* ^*flox/flox*^; *Plp* ^*CreERT2*^ mice (icKO 10 months PTI, B) and *Cdc42ep1* ^*flox/flox*^; *Cdc42ep2* ^*flox/flox*^; *Cnp* ^*Cre/wt*^ mice (dcKO at age 1 year, D) compared to respective control mice (A,C). This phenotype was not quantified; shown are electron micrographs from one mouse per condition representative of *n* = 3 mice per condition. For quantification of myelin outfoldings in optic nerves see figures 2D, 4I. Myelin outfoldings highlighted in red; asterisks indicate associated axons.
**Figure S3:** Generation of the Cdc42ep1flox allele using CRISPR/Cas9.
A Scheme showing the targeted region of the *Cdc42ep1* gene on mouse chromosome 15 (black), homology‐directed repair (HDR) template (green), introns (stippled lines), exons 2 and 3 (gray), open reading frame (bordeaux), and loxP sites (orange). Exon 2 comprises the translation initiation site (ATG, turquoise); exon 3 contains the translation termination site (Stop, blue). Genotyping primer numbers are in purple color and refer to primer sequences given in the methods section. Scale bar, 1000 bp.
B Sequences of two single guide RNAs (sgRNAs) that were designed to target intronic regions flanking exon 2 of the *Cdc42ep1 gene*. Cdc42ep1‐sgRNA1 targets intron 1 upstream of exon 2; *Cdc42ep1*‐sgRNA2 targets intron 2 downstream of exon 2. Protospacer sequences (5′ → 3′) and corresponding protospacer adjacent motifs (PAM) are indicated.
C Sequence of the *Cdc42ep1* HDR template, a 3524 bp double‐stranded DNA fragment containing two loxP sequences flanking exon 2 of *Cdc42ep1*. LoxP sites are highlighted in bold blue lettering; Exon 2 and the 5′‐end of Exon 3 are in bold black lettering. Coding sequences are underlined.

**Table S1:** Label‐free quantification of proteins in CNS myelin fractions from *Cdc42* icKO and control mice.
Identification and quantification data of proteins detected in myelin by MS^E^ (sheet 1) and UDMS^E^ (sheet 2). For each of the two conditions, tryptic peptides derived from two technical replicates (replicate digestion) per each of three biological replicates were analyzed by LC–MS (12 runs in total). Proteins (FDR < 1%; two peptides/protein) and peptides (FDR < 1%; ≥ 6 amino acids) were identified by database search against the UniprotKB/SwissProt mouse database using PLGS. Data were post‐processed with the software package ISOQuant to calculate absolute in‐sample amounts for each detected protein based on the TOP3 approach. Reported abundance values are defined as the relative amount of each protein in respect to the sum over all detected proteins (ppm: parts per million (w/w) of total protein). Typical contaminant proteins like albumin, hemoglobins, and keratins were filtered. The relative ppm abundance of myelin‐associated proteins in each sample was adjusted to yield 1 million after removal of contaminants. The ‐log10‐transformed q‐value (column minuslog10q.mod) was plotted against the log2‐transformed fold change (column logFC) to obtain the volcano plot shown in figure 2H,H′. As no imputation of missing values was performed, proteins exclusive for only one of the conditions do not appear in the volcano plot, but are appended at the end of the list. Proteins are sorted in descending order for zdist (see column descriptions below). Criteria for statistically significant regulation were as follows: fold change of at least 1.4 (log2FC CDC42 icKO/Ctrl > |0.5|) and *q*‐value below 0.05 (minuslog10q.mod > 1.3).

**Table S2:** Label‐free quantification of proteins in CNS myelin fractions from EP1 cKO, EP2 cKO, EP1/2 dcKO, and control mice.
Identification and quantification data of proteins detected in myelin by MS^E^ (sheet 1) and UDMS^E^ (sheet 2). For each of the four conditions, tryptic peptides derived from two technical replicates (replicate digestion) per each of three biological replicates were analyzed by LC–MS (24 runs in total). Proteins (FDR < 1%; 2 peptides/protein) and peptides (FDR < 1%; ≥ 6 amino acids) were identified by database search against the UniprotKB/SwissProt mouse database using PLGS. Data were post‐processed with the software package ISOQuant to calculate absolute in‐sample amounts for each detected protein based on the TOP3 approach. Reported abundance values are defined as the relative amount of each protein in respect to the sum over all detected proteins (ppm: parts per million (w/w) of total protein). Typical contaminant proteins like albumin, hemoglobins, and keratins were filtered. The relative ppm abundance of myelin‐associated proteins in each sample was adjusted to yield 1 million after removal of contaminants. For the dataset CDC42EP1/2 dcKO vs. Ctrl, the ‐log10‐transformed q‐value (column minuslog10q.mod) was plotted against the log2‐transformed fold change (column logFC) to obtain the volcano plot shown in figure 6B,B′. As no imputation of missing values was performed, proteins exclusive for only one of the conditions do not appear in the volcano plot, but are appended at the end of the list. Proteins are sorted in descending order for zdist (see column descriptions below). Criteria for statistically significant regulation were as follows: fold change of at least 1.4 (log2FC CDC42EP1/2 dcKO/Ctrl > |0.5|) and q‐value below 0.05 (minuslog10q.mod > 1.3).

## Data Availability

All relevant data are included in the main paper or files. Raw data are available from the corresponding author upon reasonable request. The mass spectrometry proteomics data have been deposited to the ProteomeXchange Consortium via the PRIDE (https://www.ebi.ac.uk/pride) (Perez‐Riverol et al. 2025) partner repository with dataset identifier PXD066943.
