# Supplementary material for: CDC42‐Effector Proteins Regulate Higher Order Structure of Septins Required for CNS Myelin Integrity
Source: Glia. 2026 Jan 6;74(3):e70134. doi: 10.1002/glia.70134 (PMC12858042; doi:10.1002/glia.70134)
Supplement: Supplementary file 1 — Figure S1: Oligodendrocytes express Cdc42, Cdc42ep1, and Cdc42ep2 according to bulk RNA‐Seq data of immunopanned cells. RNA‐Seq of cells immunopanned from mouse cortices shows that Cdc42, Ccd42ep1, and Ccd42ep2 transcripts are detected in both newly formed oligodendrocytes and myelinating oligodendrocytes. Re‐analysis of data from (Zhang et al. 2014). Mean +/‐SEM; datapoints represent individual experiments. FPKM, fragments per kilobase of transcript per million fragments mapped. Figure S2: Myelin outfoldings in the spinal cord when oligodendrocytes lack Cdc42 or both Cdc42ep1 and Cdc42ep2. A‐D Representative electron micrographs of cross‐sectioned spinal cords show that myelin outfoldings are the main pathology in spinal cords of Cdc42 flox/flox ; Plp CreERT2 mice (icKO 10 months PTI, B) and Cdc42ep1 flox/flox ; Cdc42ep2 flox/flox ; Cnp Cre/wt mice (dcKO at age 1 year, D) compared to respective control mice (A,C). This phenotype was not quantified; shown are electron micrographs from one mouse per condition representative of n = 3 mice per condition. For quantification of myelin outfoldings in optic nerves see figures 2D, 4I. Myelin outfoldings highlighted in red; asterisks indicate associated axons. Figure S3: Generation of the Cdc42ep1flox allele using CRISPR/Cas9. A Scheme showing the targeted region of the Cdc42ep1 gene on mouse chromosome 15 (black), homology‐directed repair (HDR) template (green), introns (stippled lines), exons 2 and 3 (gray), open reading frame (bordeaux), and loxP sites (orange). Exon 2 comprises the translation initiation site (ATG, turquoise); exon 3 contains the translation termination site (Stop, blue). Genotyping primer numbers are in purple color and refer to primer sequences given in the methods section. Scale bar, 1000 bp. B Sequences of two single guide RNAs (sgRNAs) that were designed to target intronic regions flanking exon 2 of the Cdc42ep1 gene. Cdc42ep1‐sgRNA1 targets intron 1 upstream of exon 2; Cdc42ep1‐sgRNA2 targets intro [file GLIA-74-0-s003.pdf]

Supplemental Figures for

Hümmert et al.

CDC42-effector proteins regulate higher order structure of septins required for CNS myelin integrity

Supplemental Figure S1.
Oligodendrocytes express Cdc42, Cdc42ep1 and Cdc42ep2 according to bulk RNA-Seq data of immunopanned cells.

Supplemental Figure S2.
Myelin outfoldings in the spinal cord when oligodendrocytes lack *Cdc42* or both *Cdc42ep1* and *Cdc42ep2.*

Supplemental Figure S3.
Generation of the Cdc42ep1^{flox} allele using CRISPR/Cas9.

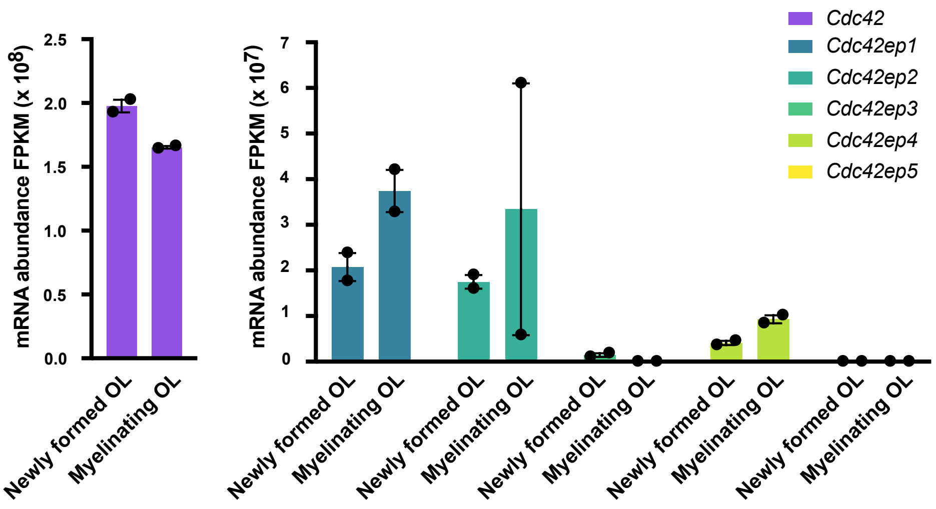

Supplemental Figure S1. Oligodendrocytes express Cdc42, Cdc42ep1 and Cdc42ep2 according to bulk RNA-Seq data of immunopanned cells. RNA-Seq of cells immunopanned from mouse cortices shows that *Cdc42*, *Ccd42ep1,* and *Ccd42ep2* transcripts are detected in both newly formed oligodendrocytes and myelinating oligodendrocytes. Re-analysis of data from (Zhang et al., 2014). Mean +/-SEM*;* datapoints represent individual experiments. FPKM, fragments per kilobase of transcript per million fragments mapped.

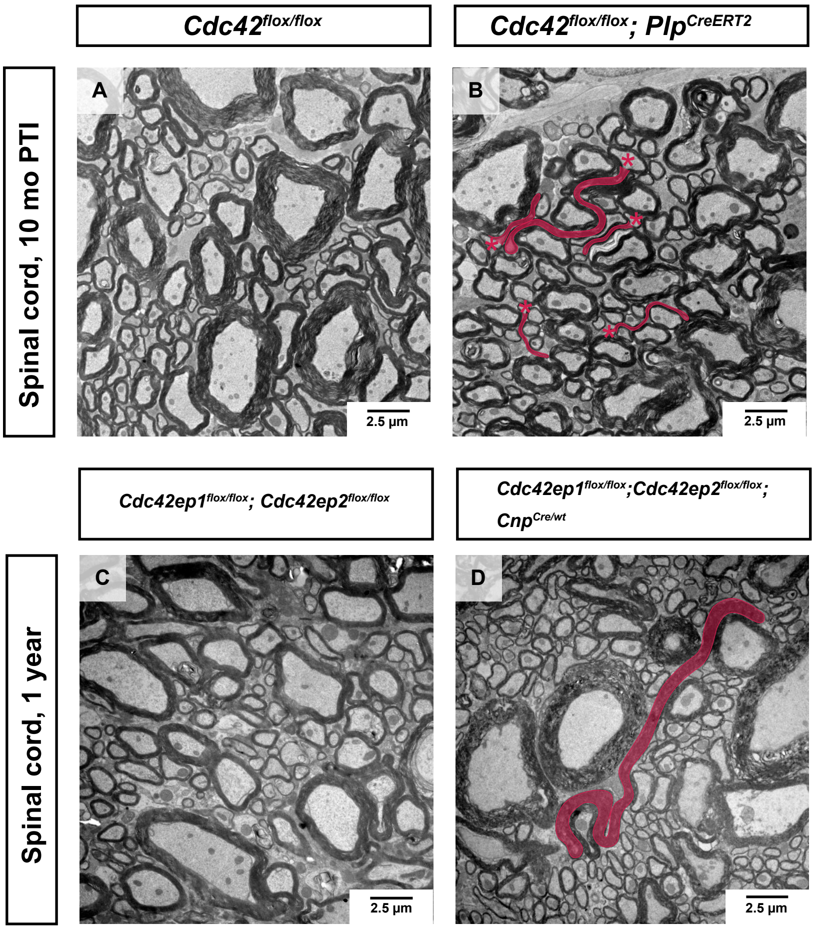

Supplemental Figure S2. Myelin outfoldings in the spinal cord when oligodendrocytes lack *Cdc42* or both *Cdc42ep1* and *Cdc42ep2.* A-D Representative electron micrographs of cross-sectioned spinal cords show that myelin outfoldings are the main pathology in spinal cords of *$Cdc42^{flox/flox}$;$Plp^{CreERT2}$* mice (icKO 10 mo PTI, **B**) and *$Cdc42ep1^{flox/flox}$;$Cdc42ep2^{flox/flox}$;$Cnp^{Cre/wt}$* mice (dcKO at age 1 year, **D**) compared to respective control mice **(A,C)**. This phenotype was not quantified; shown are electron micrographs from one mouse per condition representative of n=3 mice per condition. For quantification of myelin outfoldings in optic nerves see **Figure 2D,4I**. Myelin outfoldings highlighted in red; asterisks indicate associated axons.

A

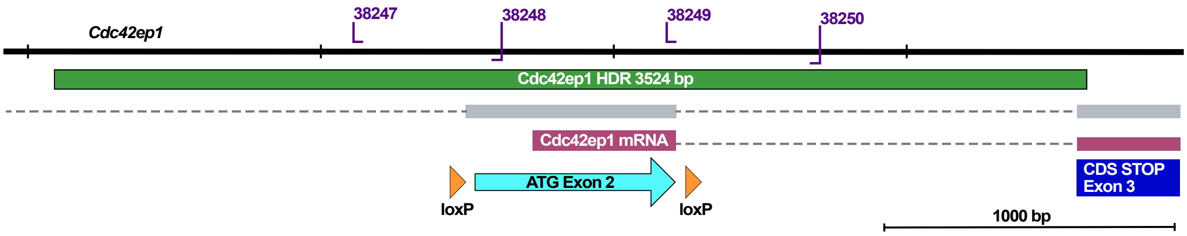

B

Name	Protospacer Sequence (5′ → 3′)	PAM	Target Region
Cdc42ep1-sgRNA1	TCAAGGACCTGTGCGTCCTT	GGG	Intron 1, upstream of Exon 2
Cdc42ep1-sgRNA2	GGAAGTTGGGCCCCCAGTCT	AGG	Intron 2, downstream of Exon 2

C

```
5′ → 3′:
accgagggaccagctgagacagtaaggacaagagagagggtccttgagggatcaggaagggtcctttggcacccacaggactc
cccccacccctccaaaacttgttccccaacaaggctcctctatcaagctgacaaacagataaagccctttcccgcaggccttc
tcagagccttgagtcaactcaagcatgtaggagccagctggaaaaagccacttctgaccgcaggattccttttccaggaagca
ccttggaggccaaggtttctgtgcaccatcagtgctggccggctgggaggccggctgtgtacacagccctgcacaggaccttg
gcagggtgccccctttctcctgacctctccaggtagaatagattgaacctacagaaaactgaaacttgccagccagaggcgat
gttgcctgggaccctgggataggtagcccaatttccctgctccattcctcactaagacgccacaggcactaagtcaccagaga
ttctcattcctgctctggcgcccgcctcctgcaggtctagtgtccacacgtcatctgacttgtgcaggagagccgtgggcacc
tccccggcgggccttaggtagagatcgatcaatcatttttctgcttcgctgatgcccgctgtgaacgcaaaacagaatggagt
ctttgtgcccgggccattcagtgatactcagaggctgccgtccactctgtgtgttctcagactctggtgtgggagtgcacgtg
tccaggtgctcacagaagccagtggaggacattggatgccttgttctgtcactctcacttagtccctggagacagagtggctg
actgaacctgaaacttgtgggtttttgtttttgtttttttttttaagctagactacctataatcctcctgtctccgttcttcc
ccctcccccccaaaacactggaatcacaggcacatgcaactatggccaacttccacgtgggtattggggaatgaaactcaggt
cctcagacatgctcagtgagcattccctcattcggtcacacacacacactcccagctctttttttgaacttctttttttgtttgt
ttggtttttttggtttttttgagacagagtttctctgtatagccctggctgtcctggaactcactttgtagaccaggctggcc
tcgaactcagaaatccgcctgcctctgtctcctgagtgctgggattaaaggcgtgcgccaccacgcccagctcctttgtgtgt
tctaatcaaggacctgtgcATAACTTCGTATAATGTATGCTATACGAAGTTATgtccttgggcagccttgtttgttctctcgg
ttgctactgtttggactggggtttcctcttctgctgacatgccacaatgctgtctttctggccttccagGTCTCCACTCCAGG
GTAGGGATCCTTTTAAGGCCCTTCTCCTGCCACCTTTGATCAGCCTCACTAGGACCAGAAGCAGAGAGAGAACCCACAGACTC
CCTCAGCCTCAGCACTGGGGACAGCGCCTTAGTAAAGTCAAGGGTCGCCTTGTGCTCCGCCATCTTCATTGAGAGGAAGGGGC
TGAGAAGGATCCCTGAGTCCAGCCTGCCATCTTCCCTGGCCCCAAGGTGGCATGGACTAGCAGTTGTAAGCAATCAAAGCTGA
TGCCCGGTCCCCAGGGGGGCACAGGAGCCCCCACCATGAGCCTGGGCAAGCTGTCTCCTGTGGGATGGGTGTCCAGTTCCCAT
GGAAAGAGACGCCTGACAGCAGACATGATCAGCCCCCCACTTGGGGACTTCCGCCACACCATGCACGTAGGCCGTGGTGGGGA
TGTCTTCGGAGATACGTCCTTCCTCAGCAACCACGGAGGCCGCTCTGGAAACACCCACCGCTCACCCCGAAGTTTCCTGGCCA
GGAAGCTCCAACAGGTTCGCAGAGTCGGGGTGCCACCTCGAAGGATGGCTTCCCCGGCTGCACCATCACCTGCTCCGCCTCCC
ATCTCGCCCATCATCAAGAACGCCATCTCCCTGCCTCAGCTCAACCAGGCCACCTATGACAGTCTGGTGATGGGCAAGCTTAG
TTTTGACAGCACGCCGGCTAGCTCCACAGATGGCCACTCTGGTTACGgtgaggaactggggtcacggcagcccagagagattc
tgccaagcctccatatcagtcttcccccacagctgtttaatctcccagtggaagttgggccccATAACTTCGTATAATGTATG
CTATACGAAGTTATcagtctaggctgaggatgaagggtgtaggcttagggaggtggggtatcccagcctgcttaggccttata
acacagaggctgaggtagaagtgatgtttgcagcttacatttctctaactctctctgtggctcacacaagtctttttcccatc
tctgggctgctctgtggctagacctccccttcctggctcctaaccagtgtcagaggttatcatggcgaggctctgagcccact
ctgatttctagtcattgttcctctgactgcagtaagcacatggactcccaagctccaatcctcacctataaaggtcccttagt
ctcattgttaagattcaatgaagtcagccggggcatggtggtgcacgcctttaatcccagcactcgggaggcagaggcaggcg
gatttctgagttcgaggccagcctggtctacaaagtgagttccaggacagccagggctataaagagaaaccctgtctcaaaaa
aaaaaaaaaaaagattcaatgaagtcatgagtccagtctgatctgcatagtgagtttcagaatagcctgggctacttactaca
ttctgagatcctgtcgcaaaatgcaaaacaaaacgaagatagagtgaaatcacacacacactgggctgctttgcagcaggtgc
caggggcagaagctgttatttgaacaaaggctcctgtaacttggcaagttgacccaggtgtgaatgccacggaggttttggca
ggacaactcaccaggggaccatgggtaacttctgccccctcgtggccatgtaatgcattacagtcttgctgccactataggca
caaggaactccatcacctgtcaggcctgggagtcctggaagcgagagttgggcaggtaaactttggaagacacaagcctgtc
ctgccagcctgtgatttctctgttctggccctggcttctggctctttcctgtatctacagatcacctcaggtgctttgagttt
acctgtagcaatcagtcggcatcagaaccaggaaccatgacccagtccacaccagggactgagtgtcaagattcaggttaggg
ttagtttcacagagagagtatagaaggctgtggggtccttacattagaaaagcaggcaaaatggaggccagcaggtgtgtgtg
tctgtgtgtgtacagcttaccaagggttttcctgactctttgtctccaagaacagattagggaccccacccctgaccttctcc
ccatttctctgctagGCCTGGAGTCTGGCTTCTGCACC
```

Supplemental Figure S3. Generation of the *Cdc42ep1*^{flox} allele using CRISPR/Cas9. A Scheme showing the targeted region of the *Cdc42ep1* gene on mouse chromosome 15 (black), homology-directed repair (HDR) template (green), introns (stippled lines), exons 2 and 3 (grey), open reading frame (bordeaux), and loxP sites (orange). Exon 2 comprises the translation initiation site (ATG, turquoise); exon 3 contains the translation termination site (Stop, blue). Genotyping primer numbers are in purple colour and refer to primer sequences given in the methods section. Scale bar, 1000 bp.

B Sequences of two single guide RNAs (sgRNAs) that were designed to target intronic regions flanking exon 2 of the *Cdc42ep1 gene.* Cdc42ep1-sgRNA1 targets intron 1 upstream of exon 2; *Cdc42ep1*-sgRNA2 targets intron 2 downstream of exon 2. Protospacer sequences (5′ → 3′) and corresponding protospacer adjacent motifs (PAM) are indicated.
C Sequence of the *Cdc42ep1* HDR template, a 3524 bp double-stranded DNA fragment containing two loxP sequences flanking exon 2 of *Cdc42ep1.* LoxP sites are highlighted in bold blue lettering; Exon 2 and the 5‘-end of Exon 3 are in bold black lettering. Coding sequences are underlined.
